# *Porphyromonas gingivalis* Evasion of Autophagy and Intracellular Killing by Human Myeloid Dendritic Cells Involves DC-SIGN-TLR2 Crosstalk

**DOI:** 10.1371/journal.ppat.1004647

**Published:** 2015-02-13

**Authors:** Ahmed R. El-Awady, Brodie Miles, Elizabeth Scisci, Zoya B. Kurago, Chithra D. Palani, Roger M. Arce, Jennifer L. Waller, Caroline A. Genco, Connie Slocum, Matthew Manning, Patricia V. Schoenlein, Christopher W. Cutler

**Affiliations:** 1 Department of Periodontics, Georgia Regents University, Augusta, Georgia, United States of America; 2 Department of Medicine, University of Colorado, Aurora, Colorado, United States of America; 3 School of Dental Medicine, Stony Brook University, Stony Brook, New York, United States of America; 4 Department of Oral Health and Diagnostic Sciences, Georgia Regents University, Augusta, Georgia, United States of America; 5 Department of Biostatistics and Epidemiology, Georgia Regents University, Augusta, Georgia, United States of America; 6 Department of Medicine, Section of Infectious Diseases, Boston University School of Medicine, Boston, Massachusetts, United States of America; 7 Department of Microbiology, Boston University School of Medicine, Boston, Massachusetts, United States of America; 8 Department of Cellular Biology and Anatomy, Georgia Regents University, Augusta, Georgia, United States of America; University of Toronto, CANADA

## Abstract

Signaling via pattern recognition receptors (PRRs) expressed on professional antigen presenting cells, such as dendritic cells (DCs), is crucial to the fate of engulfed microbes. Among the many PRRs expressed by DCs are Toll-like receptors (TLRs) and C-type lectins such as DC-SIGN. DC-SIGN is targeted by several major human pathogens for immune-evasion, although its role in intracellular routing of pathogens to autophagosomes is poorly understood. Here we examined the role of DC-SIGN and TLRs in evasion of autophagy and survival of *Porphyromonas gingivalis* in human monocyte-derived DCs (MoDCs). We employed a panel of *P. gingivalis* isogenic fimbriae deficient strains with defined defects in Mfa-1 fimbriae, a DC-SIGN ligand, and FimA fimbriae, a TLR2 agonist. Our results show that DC-SIGN dependent uptake of Mfa1+*P. gingivalis* strains by MoDCs resulted in lower intracellular killing and higher intracellular content of *P. gingivalis*. Moreover, Mfa1+*P. gingivalis* was mostly contained within single membrane vesicles, where it survived intracellularly. Survival was decreased by activation of TLR2 and/or autophagy. Mfa1+*P. gingivalis* strain did not induce significant levels of Rab5, LC3-II, and LAMP1. In contrast, *P. gingivalis* uptake through a DC-SIGN independent manner was associated with early endosomal routing through Rab5, increased LC3-II and LAMP-1, as well as the formation of double membrane intracellular phagophores, a characteristic feature of autophagy. These results suggest that selective engagement of DC-SIGN by Mfa-1+*P. gingivalis* promotes evasion of antibacterial autophagy and lysosome fusion, resulting in intracellular persistence in myeloid DCs; however TLR2 activation can overcome autophagy evasion and pathogen persistence in DCs.

## Author Summary

Among the most successful of human microbes are intracellular pathogens. By entering the intracellular milieu, these pathogens are protected from harsh environmental factors in the host, including the humoral and cellular immune responses. *Porphyromonas gingivalis* is an opportunistic pathogen that colonizes the oral mucosa and accesses the bloodstream and distant sites such as the blood vessel walls, brain, placenta and other organs. Still unclear is how *P. gingivalis* traverses from oral mucosa to these distant sites. Dendritic cells are highly migratory antigen presenting cells that “patrol” the blood, skin, mucosa and all the major organ systems. Capture of microbes by dendritic cells activates a tightly regulated series of events, including directed migration towards the secondary lymphoid organs, where processed antigens are ostensibly presented to T cells. Autophagy is now recognized as an integral component of microbial clearance, antigen processing and presentation by dendritic cells. We report here that *P. gingivalis* is able to subvert autophagic destruction within dendritic cells. This occurs through its glycoprotein fimbriae, called Mfa-1, which targets the C-type lectin DC-SIGN on dendritic cells. The other major fimbriae on *P. gingivalis*, FimA, targets TLR2, which promotes autophagic destruction of *P. gingivalis*. We conclude that DC-SIGN-TLR2 crosstalk determines the intracellular fate of this pathogen within dendritic cells, and may have profound implications for the treatment of many chronic diseases involving low-grade infections.

## Introduction

Antimicrobial autophagy or xenophagy plays an important role in controlling bacterial infection and promoting innate immunity. Recent evidence has revealed critical roles for autophagy in the ability of immune cells to recognize and selectively target microbes for elimination. [1–4][1]. Dendritic cells (DCs) are innate immune cells that serve as a bridge to the adaptive immune response. DCs capture a wide variety of microbes in the peripheral tissues for which they are equipped with broad spectrum of pattern recognition receptors (PRRs). The major classes of PRRs expressed by DCs include Toll-like receptors (TLRs), NOD-like family receptors, CARD helicases and C-type lectin receptors [5,6]. Many of the PRRs come equipped with unique phagocytic machinery evolved for efficient antigen processing and presentation [7–9]. Phagocytosis relies on a network of endocytic vesicles such as early endosomes and/or autophagosomes, which fuse with lysosomes for degradation [10]. Intracellular vesicle maturation does not necessarily proceed through similar steps in different phagocytic cells [11]. Moreover, different phagosomal maturation pathways have been reported in the same cell type [11,12]. These different pathways are dictated primarily by the initial recognition step by PRRs and by the cargo contained in the vesicle [13]. Hence the immune cell type, the PRRs engaged and the properties of the microbe seem to be crucial for microbial clearance by autophagy.

DC-SIGN (DC specific ICAM-3 grabbing non-integrin) is a C-type lectin receptor involved in pathogen uptake, signaling and antigen presentation in DCs [14–16]. For uptake DC-SIGN contains internalizing motifs in its cytoplasmic tail [17]. Interestingly, DC-SIGN has been implicated in immune suppression and regulation in certain contexts [17–19]. Most notably DC-SIGN is targeted for immune escape by several pathogens such as HIV, hepatitis C virus, herpesvirus 8 (HHV-8), *Mycobacterium tuberculosis*, *Helicobacter pylori* and *Streptococcus pneumonia* [17,20–22]. Recently, we reported that DC-SIGN engagement by the minor fimbriae (Mfa1) of *Porphyromonas gingivalis* yields weak DC maturation and an immunosuppressive cytokine profile. In the absence of Mfa1, *P. gingivalis* yields a very different DC response with high levels of IL-23 and IL-6 as well as induction of a Th1/Th17 type response [14,23]. Furthermore, this study demonstrated that the anaerobe *P. gingivalis* survives within DCs in an aerobic atmosphere, while it dies rapidly in the absence of DCs [14]. Early study of the relationship of fimbrial strain differences to alveolar bone loss, showed that this Mfa1^+^Pg strain (DPG3) induced higher bone loss than Pg381 strain in a periodontitis mouse model [24]. The destruction induced by Mfa1^+^Pg was similar to wild type strain *P. gingivalis* ATCC 53977 that has been reported to be invasive in the abscess model [25].

*P. gingivalis* expresses a number of virulence factors that bind to and signal through PRRs. The adhesion proteins, known as fimbriae, on *P. gingivalis* signal through PRRs, and facilitate invasion of host cells. *P. gingivalis* expresses both minor (MFa1) and major fimbriae (FimA) which are highly regulated depending on growth conditions [26,27]. We have previously shown that expression of Mfa1 is involved in targeting DC-SIGN while other studies have shown expression of FimA targets a non-DC-SIGN route, mostly through TLR2 [28,29]. The engagement of DC-SIGN and TLRs activates distinct signaling pathways [6,30] and we propose that differential signaling through these distinct PRRs results in differential intracellular routing and processing of *P. gingivalis* within DCs. TLRs are essential for phagosome maturation and subsequent bacterial clearance [31,32]. TLR signaling is also involved in the maturation of autophagosomes [33]. The ability of *P. gingivalis* to manipulate DC-SIGN and TLR signaling through differential fimbrial expression [26,34], could have profound effects on bacterial survival[26]. However, the role of *P. gingivalis* major and minor fimbriae in DC-SIGN-TLR2 crosstalk and its influence on survival of *P. gingivalis* within DCs has not been examined.

In the present study, a combination of approaches was used to address the role of DC-SIGN and TLRs in intracellular routing and survival of *P. gingivalis*, including blocking PRRs and autophagy, siRNA gene silencing and activation of autophagy in monocyte derived DCs (MoDCs), To address the role of fimbriae in this regard we utilized defined bacterial mutants, that solely express minor fimbriae (Mfa1^+^Pg), major fimbriae (FimA^+^Pg) or are deficient in both fimbriae (MFB) [35] (Table 1). Our results indicate that engagement of DC-SIGN by MFA-1 allows *P. gingivalis* to evade autophagy and lysosome fusion, resulting in pathogen persistence and survival within DCs. In contrast, activation of autophagy or of TLR2 by *P. gingivalis* expressing FimA results in autophagy mediated killing of this pathogen within DCs. Collectively, our studies reveal a novel mechanism that enables this pathogen to evade host detection and clearance and which could have profound implications for the treatment of other diseases involving low-grade chronic infection.

**Table 1:** *P. gingivalis* wild type and isogenic fimbriae deficient mutants.

Strain	Description	Predominant receptor targeted on DCs [14,23] [28,29]
Pg381	Wild-type (381), which expresses both minor (Mfa1) and major (FimA) fimbriae.	DC-SIGN and TLR2
Mfa1^+^Pg	Isogenic major fimbriae-deficient mutant DPG3, which expresses only the minor fimbriae (Mfa1).	DC-SIGN
FimA^+^Pg	Isogenic minor fimbriae-deficient mutant MFI, which expresses only the major fimbriae (FimA).	TLR2
MFB	Isogenic double fimbriae mutant MFB, which lacks both minor and major fimbriae.	TLRs

## Results

### High intracellular content of Mfa1^+^Pg within human MoDCs

**Fig 1:**
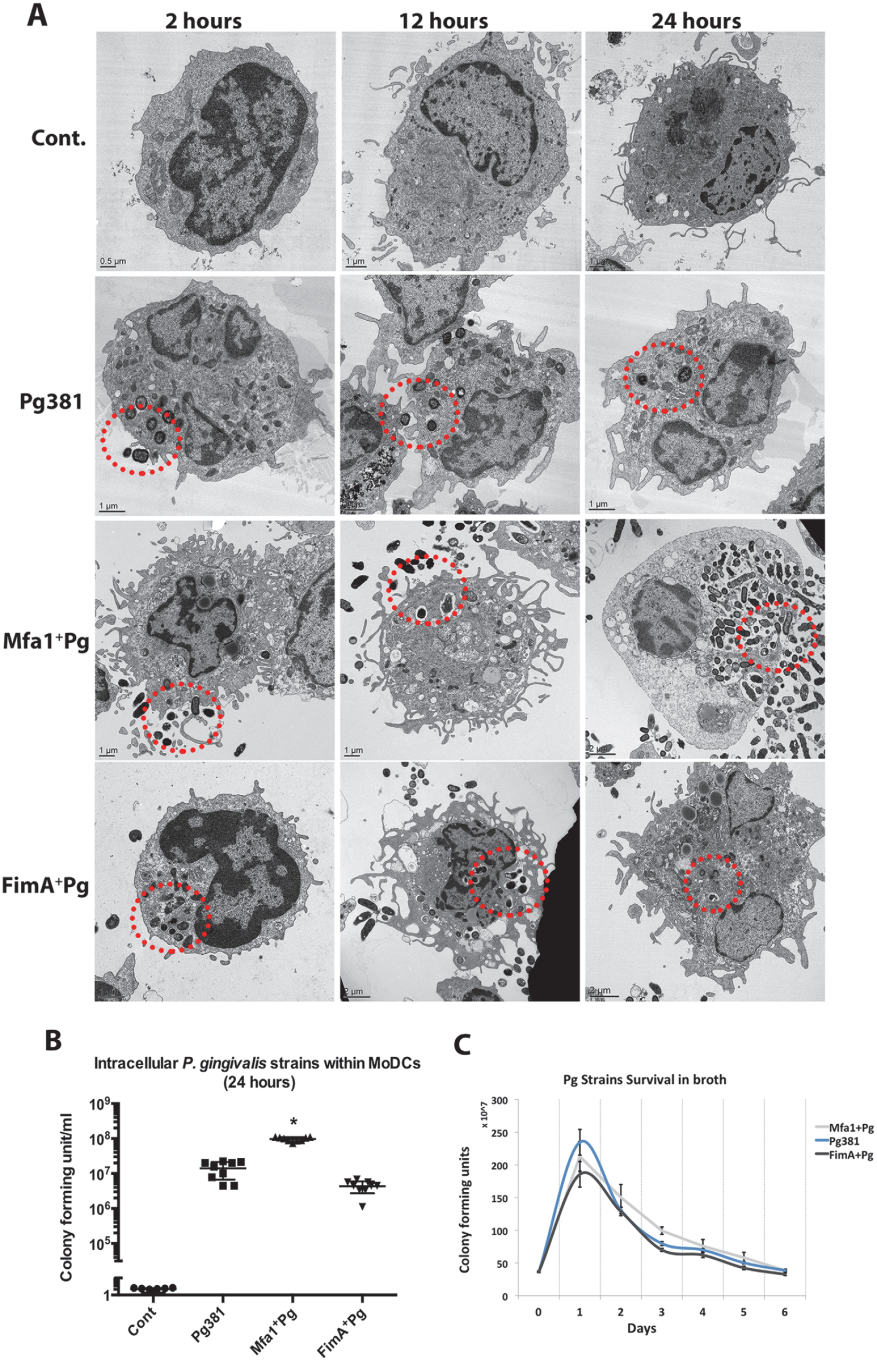
High intracellular content of Mfa1^+^Pg within human MoDCs. **A)** Transmission electron microscopy (TEM) of MoDCs infected with *P. gingivalis* for 2, 12 and 24 hours (left, middle and right panels). The sections show the intra-and extra-cellular contents of Cont. (un-infected), Pg381, Mfa1^+^Pg and FimA^+^Pg infected MoDCs for the different time points. **B)** The figure shows the intracellular content of *P. gingivalis* strains after 24 hours of infection. MoDCs infected with Pg381 or isogenic mutants strains were lysed and the survived intracellular bacteria were re-suspended and maintained in anaerobic broth for 5 days. The data represents CFU within MoDCs harvested from three healthy individuals. The means ±standard deviation (in triplicates) were analyzed by One-way ANOVA of different groups and Tukey’s test for multiple group comparisons within 3 different experiments (* statistical significance at the *p*<0.001). **C)** Growth curves of Pg381 and mutant strains (Mfa1^+^Pg and Fima^+^Pg) in anaerobic condition in the absence of DCs. The means ±standard deviations were analyzed by One-way ANOVA of different groups and Tukey’s test for multiple group comparisons within 3 different experiments.

At 2, 12 and 24h after bacterial co-culture with MoDCs, the MoDCs were imaged for intracellular *P. gingivalis* by epifluorescence microscopy and transmission electron microscopy (TEM). All *P. gingivalis* strains except the double fimbriae negative *P. gingivalis* strain MFB (Table 1) were taken up by MoDCs. There were marked differences in the *P. gingivalis* content of MoDCs at 2, 12 and 24 hours, particularly when comparing double fimbriae positive strain Pg381 to Mfa1^+^Pg (Fig. 1A). We observed a higher number of Mfa1^+^Pg within MoDCs (Fig. 1A) (S1 Fig.) This difference was most apparent after 24 hours, with large numbers of intra-and extra-cellular bacteria present. In contrast, MoDCs infected with Pg381 showed minimal bacterial content after 24 hours. Survival of intracellular bacteria was then assessed quantitatively by lysing MoDCs and growing bacteria in broth cultures and on anaerobic blood agar plates. Mfa1^+^Pg was recovered at higher numbers from MoDCs lysates in broth and on blood agar compared to Pg381 Fig. 1B). No significant difference was detected in the growth or death patterns of all strains in the media under anaerobic conditions in the absence of DCs (Fig. 1C).

### Infection of MoDCs with Mfa1^+^Pg increases the expression of DC-SIGN in MoDCs

**Fig 2:**
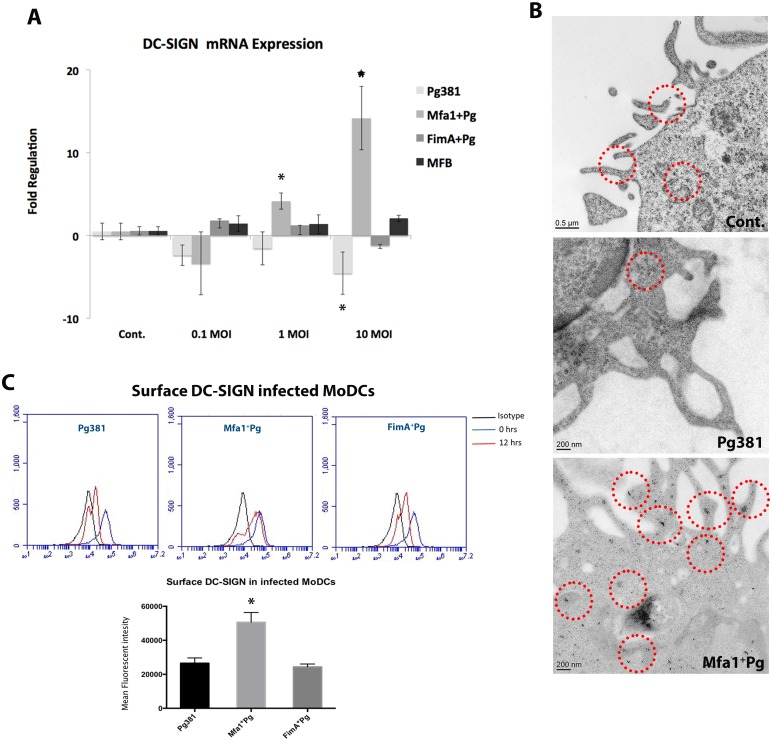
Mfa1^+^Pg up-regulate the expression of DC-SIGN in human MoDCs. **A)** DC-SIGN mRNA expression in *P. gingivalis*-infected MoDCs at 0.1, 1 and 10 MOIs. The figure shows the gene expression after 12 hours of Pg381 and mutant strains infections. The target gene (DC-SIGN) was normalized using the endogenous control GAPDH (ΔCt) and fold regulations were calculated using 2^-(ΔΔCt)^ method. The statistical analysis was performed using the *t-test*, which accounts for the clustering of infected and un-infected controls within 3 different experiments (* *p*<0.001). **B)** Immuno-electron microscopy of un-infected MoDCs (Cont.) (upper panel), MoDCs infected with Pg381 (middle panel) and Mfa1^+^Pg mutants (lower panel). Gold particles (marked with red rings) for positive DC-SIGN were detected in the cell membrane and cytoplasm of cells infected with Mfa1^+^Pg strains. Minimal positive staining for DC-SIGN was detected in the membranes of MoDCs infected with Pg381, while no cytoplasmic gold labeling was detected in these cells. **C)** Flow cytometry analysis of surface DC-SIGN in human MoDCs after infection with Pg381, Mfa1^+^Pg and FimA^+^Pg. The analysis of the intensity used Kruskal-Wallis test analysis of different groups and Dunn’s test for multiple comparisons 3 different experiments (* *p*<0.01).

To determine whether expression of DC-SIGN [14] was altered by *P. gingivalis* infection, MoDCs were infected with all the strains at different multiplicities of infection (MOIs) and gene expression of DC-SIGN was quantified at 2, 6, 12 and 24 hours (Fig. 2A) (S2 Fig.). At 12 hours, a distinct pattern of DC-SIGN expression was detected in MoDCs infected with Mfa1^+^Pg compared to Pg381 and FimA^+^Pg. Infection with Mfa1^+^Pg up-regulated DC-SIGN mRNA at 1 and 10 MOIs in a dose dependent manner (*p*<0.01) (Fig. 2A) (Table 2). In contrast, we observed decreased expression of DC-SIGN when MoDCs were incubated with Pg381 (MOI-10) down-regulated DC-SIGN mRNA expression significantly (*p*<0.05) at 12 hours (-4.55 fold). Fold regulations were calculated relative to un-infected MoDCs (Fig. 2A) (Table 2).

**Table 2:** DC-SIGN mRNA expression in MoDCs at 12 hours.

		Fold Regulation		
	Strain	Avg.	SD.	*P-value*
0.1 MOI	Pg381	-2.4	1.2	0.063
	Mfa-1^+^Pg	-4.6	5.8	0.219
	FimA^+^Pg	1.8	0.2	0.096
	MFB	1.4	0.9	0.068
1 MOI	Pg381	-1.6	2.0	0.181
	Mfa-1+Pg	4.2	1.0	* **0.003**
	FimA+Pg	1.2	0.0	0.148
	MFB	1.4	1.2	0.421
10 MOI	Pg381	-4.6	2.6	* **0.024**
	Mfa-1^+^Pg	14.2	3.8	* **0.006**
	FimA^+^Pg	-1.2	0.1	0.016
	MFB	2.1	0.4	0.074

Avg.: Average of the fold regulation. SD.: Standard deviation.

*Statistically significant at the *P value*<0.01 level of significance.

Comparisons of fold regulations for gene expression were considered significant if these values were at least 2 fold increased or decreased in comparison to the control (un-infected MoDCs) with *P value*<0.01.

We also examined DC-SIGN expression on MoDCs by immunoelectron microscopy (Fig. 2B) and flow cytometry (Fig. 2C). The results confirm a difference in DC-SIGN expression in MoDCs as a function of *P. gingivalis* strain. Mfa1^+^Pg induced higher positive immuno-labeling for DC-SIGN in MoDCs relative to Pg381 (Fig. 2B). These results correlated well with our initial results from the mRNA analysis. DC-SIGN was detected on the membrane but also in the cytoplasm of MoDCs infected with Mfa1^+^Pg. The presence of cytoplasmic staining is consistent with previous evidence for Mfa1^+^Pg localization to DC-SIGN positive intracellular compartments [14] and the possibility of receptor recycling to the cell membrane after the phagocytic process. The cells infected with Pg381 showed minimal staining for DC-SIGN at the cell membrane and no cytoplasmic staining was detected at any of the time points (Fig. 2B). Stably transfected DC-SIGN positive and negative Raji cells served as positive and negative controls respectively for DC-SIGN expression by immuno-electron microscopy (S3 Fig.).

We quantitatively assessed the increase of DC-SIGN in MoDCs infected with MFa1^+^Pg by flow cytometry analysis (Fig. 2C) (S4 Fig.). We also monitored the expression of other C-type lectins and TLRs on the infected MoDCs by flow cytometry. Although *P. gingivalis* up-regulated the expression of TLR2 and CXCR4, we did not observe strain specific differences in expression of these receptors (S4 Fig.). Moreover, there were no changes in the expression of DCIR and mannose receptor (MMR) upon *P. gingivalis* infection. We did observe increased expression of Dectin receptors, but only on Mfa1^+^Pg infected MoDCs (S4 Fig.).

### Difference in early but not late endosomal routing of Mfa1^+^Pg in MoDCs

To analyze the phagosomal machinery involved in uptake and routing of different *P. gingivalis* strains by MoDCs, we assessed levels of the GTPase Rab family of proteins at early (2 hours) through late (24hours) stages of infection. Furthermore, we monitored co localization of the *P. gingivalis* strains with Rab proteins at these time points. Although both Pg381 and Mfa1^+^Pg were taken up at 2 hours, only Pg381 was associated with Rab5 at significantly higher levels as compared to Mfa1^+^Pg (Table 3) (S5 Fig. A and B). Association of Pg381 with Rab5 within MoDCs was detected up to 12 hours. After this point, detectable bacteria and Rab5 signals significantly decreased at 24 hours. The Rab5 signal was weak at all time points in MoDCs infected with Mfa1^+^Pg. In addition, Mfa1^+^Pg were more apparent than within MoDCs at 24 hours (S5 Fig. A and B).

MoDCs generally showed weak staining for Rab7 following infection either with Pg381 or Mfa1^+^Pg up to 24 hours (S5 Fig. C and D). Since Rab7 was not detected during intracellular processing of any of the *P. gingivalis* strains examined, we investigated whether anti-bacterial autophagy may be involved in killing of Pg381 but not Mfa1^+^Pg.

**Table 3:** Pearson’s co-localization of Rab5 (red) and P. gingivalis (green) signals.

Strain	Pearson’s co-localization		P-value
	Avg	Sdv	
Pg381	0.8649	0.0702	
Mfa1^+^Pg	-0.2518	0.0600	* **0.0023**

The statistical analysis was performed using the t-test compared to Pg381 infected cells, 3 different experiments (*p-value <0.01). Avg.: average, Sdv: standard deviation.

### Induction of autophagy by Rapamycin inhibits the survival of Mfa1^+^Pg within MoDCs

**Fig 3:**
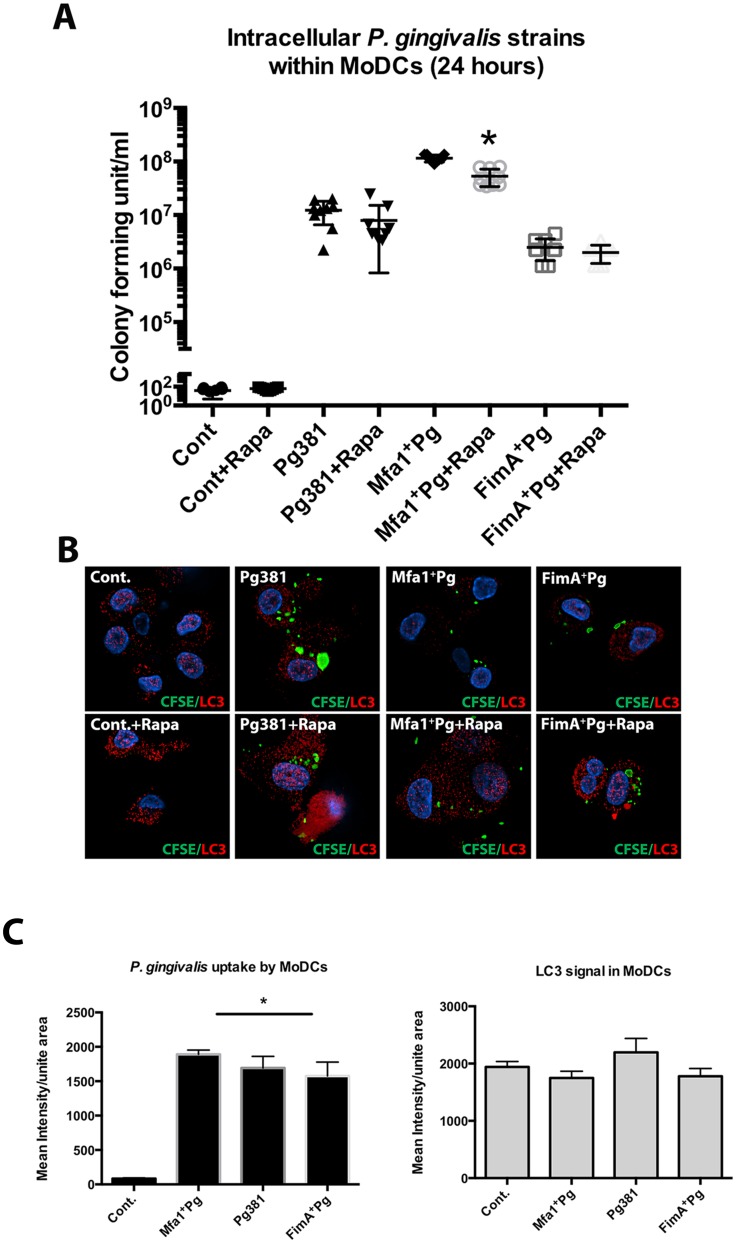
Lower intracellular content of Mfa1^+^Pg in MoDCs treated with Rapamycin. **A)** Pg381 and mutant strains count after 24 hr incubation with human MoDCs with/without Rapamycin treatment. The survived bacteria were measured after maintaining the lysed MoDCs suspension in anaerobic broth for 5 days. The plot represents the means ±standard deviation of CFU within MoDCs harvested from three healthy individuals (* *P*<0.001). The analysis of readings used One-way AVOVA analysis of different groups and Tukey’s test for multiple comparisons. **B)** Epifluorescence microscopy images of MoDCs treated with Rapamycin 1 hour after *P. gingivalis* infections. LC3-II (red-fluorescent dye) and the bacterial strains (green CFSE) were studied in MoDCs 11 hours after Rapamycin treatment (12 hours after infections). **C)** Quantifications of the fluorescent intensity of CFSE-labeled *P. gingivalis* and LC3-II signals within infected MoDCs using NIS-Elements BR software. One-way ANOVA analysis was used to compare the means of intensity of different groups and Tukey’s test for multiple comparisons (* *P*<0.001).

To investigate the role of autophagy as a putative survival mechanism utilized by Mfa1^+^Pg, the viability of *P. gingivalis* strains in MoDCs was monitored after induction of autophagy by the mTOR inhibitor, Rapamycin. Initial studies established that the viability of MoDCs and of *P. gingivalis* alone were not Rapamycin-sensitive at the concentrations used. Entry into MoDCs resulted in a significant increase in survival of Mfa1^+^Pg at 24 hours (*p*<0.001) (Fig. 3A). Rapamycin treatment of Mfa1^+^Pg-infected MoDCs (Mfa1^+^Pg+Rapa) significantly decreased *P. gingivalis* survival by ~48% (*p* <0.001) (Fig. 3A). Increased autophagy induction was confirmed by immuno-labeling of LC3-II in MoDCs treated with Rapamycin for 11 hours (1 hour after *P. gingivalis* infections). Rapamycin treatment increased the LC3-II signal in cells infected with all fimbriated strains as well as in un-infected (Cont.) (Fig. 3B and C).

**Fig 4:**
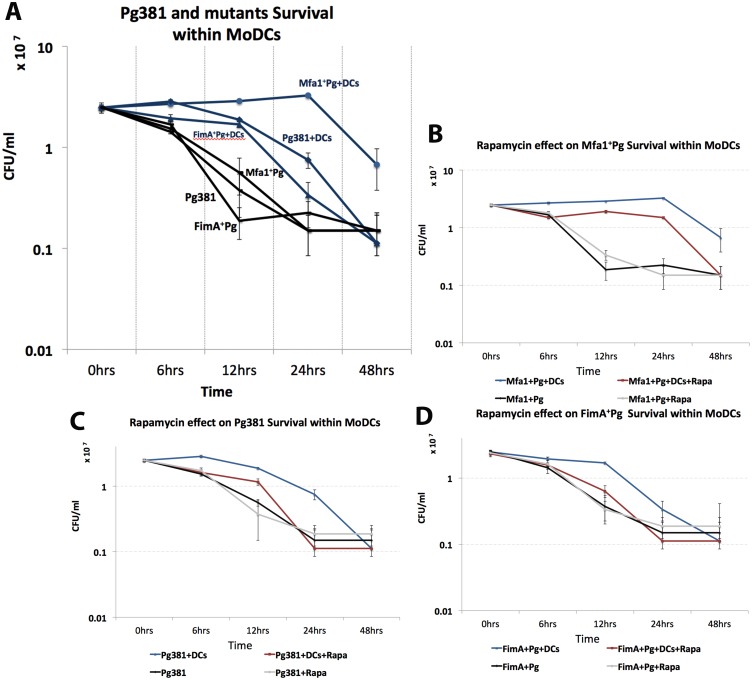
Induction of autophagy impairs the survival of Mfa1^+^Pg strain within MoDCs. **A)** Survival of *P. gingivalis* strains within MoDCs after 6, 12, 24 and 48 hours. Blue lines show *P. gingivalis* strains survival within MoDCs and their survival in anaerobic condition in the absence of DCs are showed in black lines. The effect of rapamycin on Mfa1^+^Pg, Pg381 and FimA^+^Pg survival within MoDCs are shown in figures **B, C and D** respectively. A three-factor repeated measures ANOVA using mixed models was used to test the effect of strain and rapamycin treatment over time on OD reading. The survival curves for the strains are showed in blue, while the effect of rapamycin treatments are in red. Bacterial survivals in the absence of MoDCs with and without rapamycin are plotted in grey and black, respectively. Statistical analysis showed that the strain by rapamycin treatment overtime interaction indicates the pattern of means in each strain (Mfa1^+^Pg, Pg381 and FimA^+^Pg) between treated (rapamycin) and untreated were significantly different overtime (*p-value* <0.=001).

To determine the kinetics of survival of *P. gingivalis* within MoDCs, as well as the involvement of autophagy, intracellular bacteria were monitored after 6, 12, 24 and 48 hours of incubation with MoDCs with or without Rapamycin (Fig. 4). The levels of *P. gingivalis* Mfa1^+^Pg within MoDCs (Mfa1^+^Pg+DC) were the highest at all time points except at 6 hours when we observed similar levels to that observed with Pg381+DCs (Fig. 4A). Levels of Pg381 and FimA^+^Pg within MoDCs (Pg381+DCs and FimA^+^Pg+DCs) were nonetheless significantly higher than bacteria without MoDCs, until 12 hours, at which point we observed a significant decrease in survival of both strains at 24 and 48 hours. Moreover, the highest level of Mfa1^+^Pg was observed at 24 hours, with the numbers of *P. gingivalis* increasing within MoDCs at 6, 12 and 24 hours (Fig. 4A). Activation of autophagy with Rapamycin significantly inhibited Mfa1^+^Pg survival at 6, 12 and 24 hours within MoDCs (Mfa1^+^Pg+DCs+Rapa) (Fig. 4B). However, the numbers of Mfa1+Pg detected within MoDCs treated with Rapamycin (Mfa1+Pg+DCs+Rapa) were still higher than bacteria alone (Mfa1^+^Pg) at 12 and 24 hours (Fig. 4B). For Pg381, rapamycin also significantly decreased intracellular survival, with no significant differences detected relative to bacteria alone except at 12 hours (Fig. 4C). FimA^+^Pg exhibited significant intracellular survival (FimA^+^Pg+DCs) only at 12 hours, which was significantly inhibited with rapamycin treatment (Fig. 4D). The analysis of the data using three-factor repeated measures ANOVA showed that both time and intracellular environment were significant factors (P<0.05) in *P. gingivalis* survival, with rapamycin significantly impairing *P. gingivalis* survival with MoDCs.

### Low autophagosomal marker LC3-II in MoDCs following uptake of Mfa1^+^Pg

**Fig 5:**
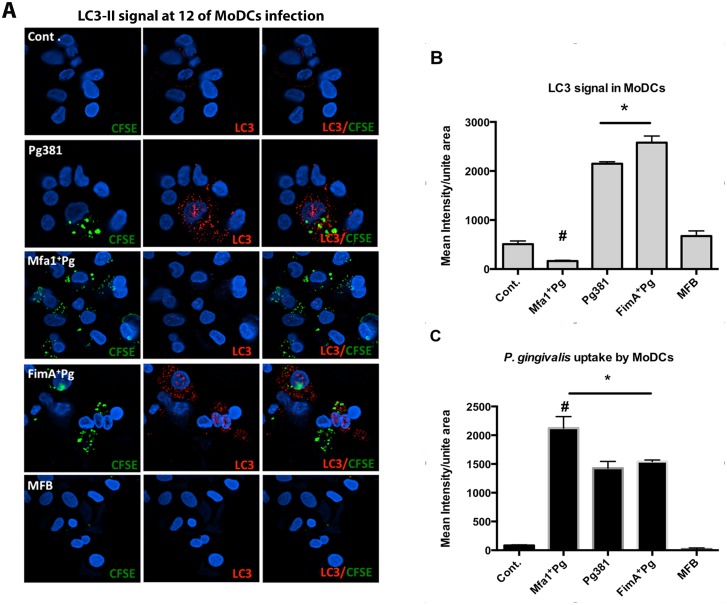
Low LC3-II signals in human MoDCs infected with Mfa1^+^Pg. **A)** Epifluorescence microscopy images of MoDCs infected with Pg381, Mfa1^+^Pg, FimA^+^Pg and MFB strains after 12 hours. LC3-II was detected in red-fluorescent (Texas red) dye and the bacterial strains were pre-labeled with CFSE (green). Co-localization of *P. gingivalis* and LC3-II showed in the right panels. **B)** Quantifications of the fluorescent intensity of LC3-II within infected MoDCs using NIS-Elements BR software. One-way ANOVA analysis was used to compare the means of intensity of different groups and Tukey’s test for multiple comparisons of three different experiments (* *p*<0.001, # *p*<0.01). **C)** Bacterial uptake by MoDCs was determined by CFSE fluorescent intensity (* *p*<0.001, # *p*<0.01).

Due the marked difference in Rab5 induction in MoDCs infected with Pg381 and Mfa1^+^Pg, and the important role of Rab5 in regulation of subsequent autophagy [36,37] we tracked the LC3-II signal in MoDCs infected with labeled *P. gingivalis* strains between 2 to 24 hours (S6 Fig. A and B). LC3-II is the active form of cytosolic LC3 that associates with the autophagosome until cargo degradation [38]. Pg381 infection resulted in significant increases of LC3-II within MoDCs at 2, 6 and 12 hours (*p* = 0.0317, 0.008 and < 0.001, respectively). In contrast, LC3-II remains unchanged in MoDCs infected with Mfa1+Pg during 24 hours of infection. The highest level of LC3-II within Pg381-infected MoDCs was evident at 12 hours (mean fluorescent intensity = 2360.06 ±251.72) (S6 Fig. A and B). Hence, further analysis of the uptake of different *P. gingivalis* strains by MoDCs and the expression of LC3-II were carried out at the 12 hr time point (Fig. 5A and B). Infection with Pg381 and FimA^+^ Pg, but not Mfa1^+^Pg increased LC3-II levels in MoDCs during the first 12 hours. (Fig. 5A) (S6 Fig. A and B). Although there was generally a low level of co-localization between all *P. gingivalis* strains with LC3-II, strains Pg381 and FimA^+^Pg showed higher Pearson’s correlation than Mfa1^+^Pg with LC3-II. Moreover, quantification of the LC3-II signals in infected MoDCs revealed significant increases in cells infected with Pg381 and FimA^+^Pg (*P<0.001* for both strains compared to Mfa1^+^Pg). Cells infected with Mfa1^+^Pg, in contrast, showed decreased levels of LC3-II compared to un-infected cells (Cont.) (Fig. 5B). Uptake of Pg381 and all mutant strains except MFB was confirmed by intensity quantification of CFSE (Fig. 5C). The highest uptake was detected in cells infected with Mfa1^+^Pg yet these cells had the lowest level of LC3-II signal (Fig. 5B and 5C).

**Fig 6:**
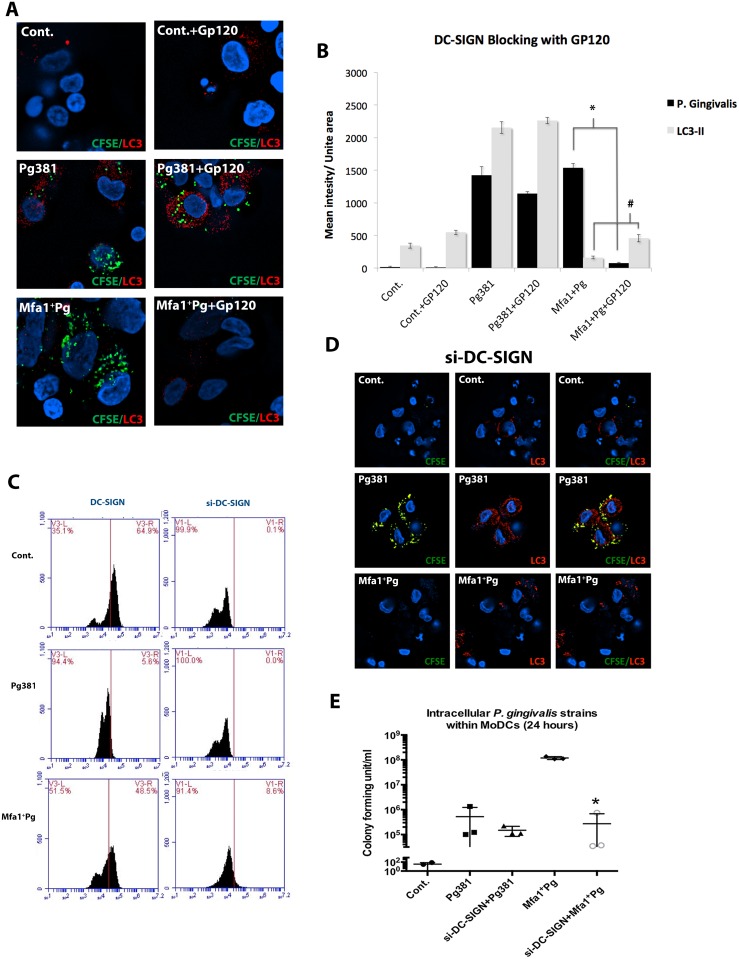
Blocking of DC-SIGN inhibits Mfa1^+^Pg uptake and restores basal LC3-II signal in MoDCs. **A)** Immuno-fluorescence images of LC3-II and *P. gingivalis* within MoDCs pre-treated with GP120 (DC-SIGN blocker) after 12 hours of infection. LC3-II was detected in red-fluorescent dye and the bacterial strains were pre-labeled with green CFSE. **B)** Fluorescent intensity of LC3-II and *P. gingivalis* strains signal (in triplicates) were statistically analyzed (* and # *p*<0.001). **C)** Flow cytometry of MoDCs treated with siRNA for DC-SIGN (si-DC-SIGN) after 12 hours. Left panels show the decrease of DC-SIGN in Cont. (un-infected), Pg381 and Mfa1^+^Pg-infected MoDCs. **D)** Epifluorescence microscopy images of MoDCs (si-DC-SIGN) infected with Pg381 and Mfa1^+^Pg. LC3-II was detected in red-fluorescent dye and the bacterial strains were pre-labeled with green CFSE. **E)** The figure shows the CFU counts of the *P. gingivalis* strains with MoDCs that lack DC-SIGN (si-DC-SIGN). The analysis of readings used One-way ANOVA analysis of different groups and Tukey’s test for multiple comparisons (* *p*<0.001).

Infection of MoDCs by Mfa1^+^Pg has previously been shown to depend on engagement of DC-SIGN [14]. We confirmed this result by blocking DC-SIGN with HIV gp120, prior to infection (Fig. 6A and 6B). Blocking DC-SIGN increased the LC3-II signal in MoDCs prior to addition of Mfa1^+^Pg (Fig. 6B). Moreover, blocking DC-SIGN restored the basal expression of LC3-II in MoDCs (Fig. 6B). To confirm the HIV gp120 blocking experiments, we additionally knocked down DC-SIGN using siRNA. DC-SIGN knockdown inhibited uptake of Mfa1^+^Pg but not Pg381 (Fig. 6C and 6D) and restored LC3-II signals in MoDCs (Fig. 6C and 6D). Furthermore, DC-SIGN knockdown significantly decreased survival of Mfa1^+^Pg in MoDCs (Fig. 6E). A scrambled sequence control did not inhibit uptake or effect LC3-II signal. To confirm the contribution of actin-mediated endocytic trafficking in LC3-II induction, MoDCs were treated with cytochalasin-D (CytD) prior to infection. CytD significantly inhibited intracellular localization of both Pg381 and Mfa1^+^Pg by MoDCs and restored the LC3-II signals to the basal level in MoDCs (S7 Fig. C and D).

**Fig 7:**
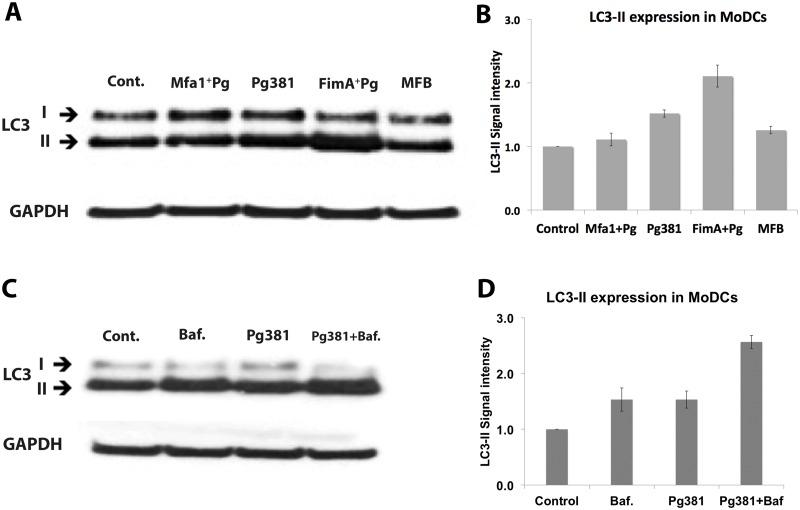
Low LC3-II expression in MoDCs infected with *P. gingivalis* expressing Mfa1. **A)** Western blot detection of LC3 in MoDCs infected with Pg381 and mutant strains for 12 hours. **B)** The mean ±standard deviation of LC3-II intensity represents three different experiments and the values were analyzed using Kruskal-Wallis test analysis of different groups and Dunn’s test for multiple comparisons. **C)** Blot shows the autophagy flux test after blocking the lysosomal fusion with Bafilomycin in MoDCs. Cells were treated with Bafilomycin for 4 hours at 4nM. **D)** LC3-II intensity in MoDCs infected with Pg381 strains with and without Bafilomycin. The analysis of the intensity of three different experiments used Kruskal-Wallis test analysis of different groups and Dunn’s test for multiple comparisons.

We further confirmed LC3-II conversion in MoDCs by Western blot analysis (Fig. 7). Pg381 and FimA^+^Pg increased LC3-II expression in MoDCs. In contrast, Mfa1^+^Pg-infected MoDCs showed no significant difference in LC3-II compared to the uninfected control (Cont.) or MFB treated MoDCs (Fig. 7A and 7B). To determine if increased LC3-II signal in MoDCs infected with Pg381 was indeed due to increased induction, as opposed to accumulation from lack of autophagosomal—lysosome fusion, the latter was inhibited by Bafilomycin in the flux test as reported [39]. Dose response of Bafilomycin was confirmed in MoDCs, with the highest LC3-II accumulation observed between 3–4nM (S7 Fig. A and B). Bafilomycin treatment further increased LC3-II by Pg381, indicating an increase in autophagy by Pg381 rather than a block in autophagosome-lysosomal fusion (Fig. 7C and 7D).

### TLR signaling increases LC3-II and inhibits the survival of Mfa1^+^Pg within human MoDCs

**Fig 8:**
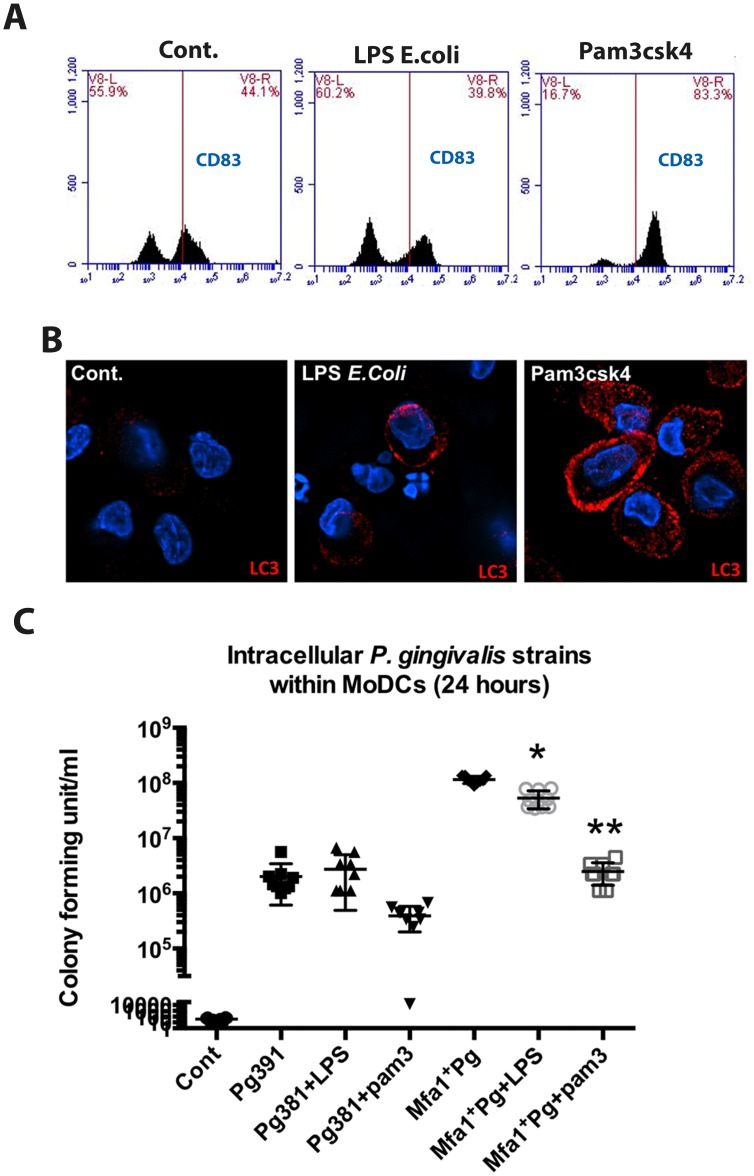
TLRs activation restores LC3-II expression and inhibits the growth of Mfa1^+^Pg within human MoDCs. **A)** Flow cytometry of CD83 on MoDCs after incubation of TLR4 ligand (*E. coli* LPS) and TLR1 and 2 ligand (Pam3csk4) for 4 hour. **B)** Immuno-fluorescence images of LC3-II (red) within MoDCs after incubation with TLR4 and TLR1&2 ligands (*E. coli* LPS and Pam3csk4) **C)** The plot represents the means ±standard deviation of CFU within MoDCs harvested from three healthy individuals after 24 hours (** *p*<0.001).

To establish the role of TLR signaling in autophagy induction leading to DC maturation and intracellular killing of *P. gingivalis*, we activated TLR pathways using specific agonists for TLR1/2 (Pam3csk4) and TLR4 (*E. coli* LPS). The results demonstrate that TLR1/2 activation was highly potent in stimulating CD83 expression and intracellular LC3-II within MoDCs infected with *P. gingivalis* (Fig. 8A and 8B). In addition, we observed a significant reduction in the Mfa1^+^Pg counts within MoDCs treated with Pam3csk4 after 24 hours of infection (Fig. 8C).

### DC-SIGN targeting Mfa1^+^Pg routed preferentially to single membrane intracellular structures within MoDCs

**Fig 9:**
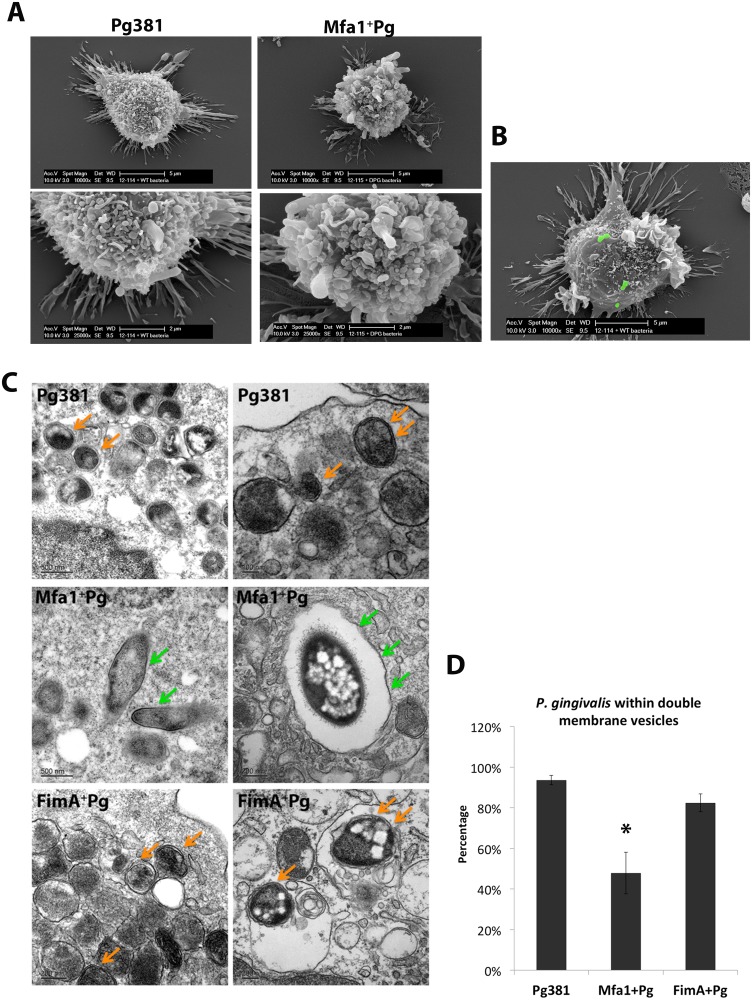
Formation of double-membrane vesicles in *P. gingivalis*-infected MoDCs. **A)** Scanning electron microscopy (SEM) of MoDCs of the early interaction of MoDCs with Pg381 and Mfa1^+^Pg (upper panel 10000x and lower panel 25000x). **B)** SEM for MoDCs interacting with Pg381 (green stains for bacteria are computer generated). **C)** Transmission electron microscopy (TEM) of autophagosome like structures within MoDCs infected with Pg381, Mfa1^+^Pg and FimA^+^Pg for 12 hours (upper, middle and lower panels, respectively). The right and left sections show the different magnifications of randomly selected sections. Pg381 and FimA^+^Pg strains are mostly enclosed in the characteristics double-membrane intracellular vesicles (Orange arrows). Contrary, Mfa1^+^Pg escaped these autophagic (double-membrane) vesicles and enclosed within single membrane structures or freely occupy the cytoplasm (Green arrows). **D)** The ratio of bacteria included in the double membrane were compared to total number of bacteria within MoDCs and plotted as percentage. Counting of the bacteria included in single or doubled membrane vesicles after 12 hours of infection. Each strain was counted in three randomly selected grids for each sample. The analysis of the bacterial counts used Kruskal-Wallis test of different groups and Dunn’s test for multiple comparisons (*p<0.01).

The initial interaction of *P. gingivalis* with the outer membrane of MoDCs was visualized by scanning electron microscopy (SEM) after 2 hours of infection (Fig. 9A and 9B). Both strains (Pg381 and Mfa1^+^Pg) were able to engage the MoDC surface at 2 hours (Fig. 9A and 9B). To directly visualize formation of double membrane autophagosomes, TEM analysis was performed in MoDCs infected with all fimbriated strains. Tracking of Pg381 and FimA^+^Pg within MoDCs after 12 hours of infections demonstrated that the majority of bacteria were contained in double membrane structures. In contrast, greater numbers of Mfa1^+^Pg were consistently detected within single membrane vesicles in the cytoplasm of MoDCs (Fig. 9C). Quantification of the double membrane structures that contained bacteria in were carried out in three randomly selected EM grids of each sample. The ratio of Mfa1^+^Pg trapped in double membrane relative to the total intracellular bacteria was significantly lower than Pg381 and FimA^+^Pg (Fig. 9D).

### Low level of lysosomal marker (LAMP1) in MoDCs after DC-SIGN mediated uptake of *P. gingivalis*

**Fig 10:**
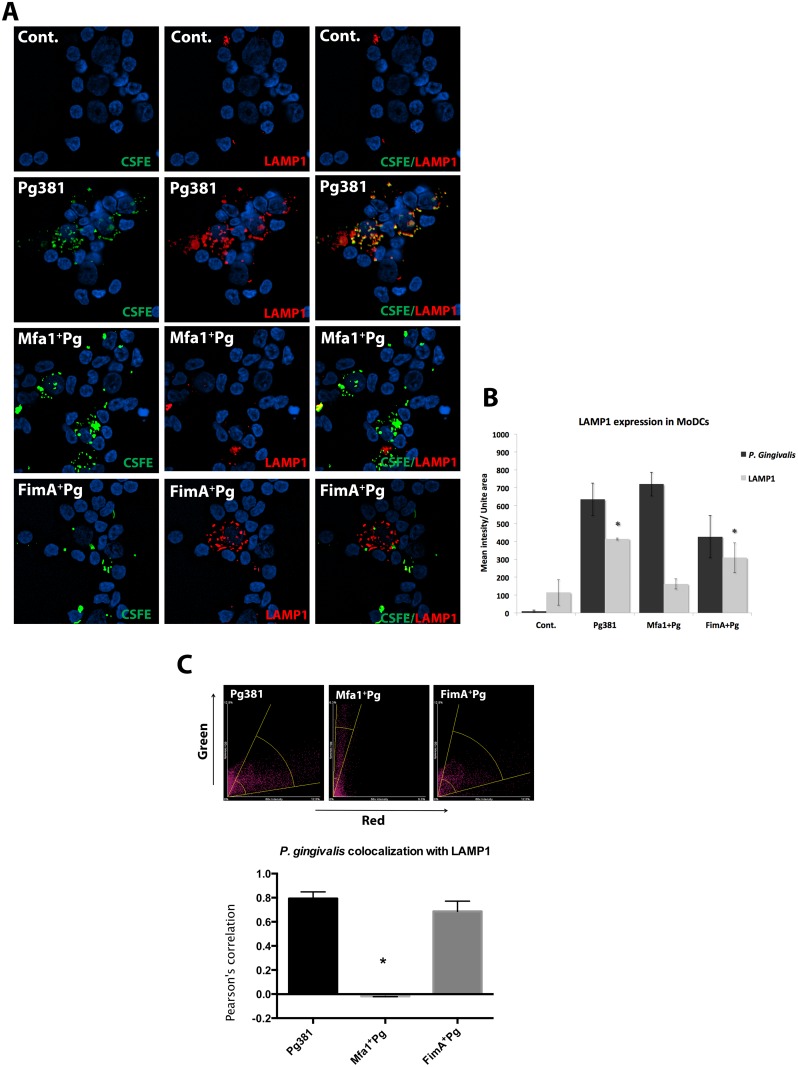
LAMP1 decreased within MoDCs infected with *P. gingivalis* expressing Mfa1. **A)** Epifluorescence microscopy images of MoDCs infected with pre-labeled bacteria (green-CFSE). LAMP1 detected by transduction of red fluorescent protein (RFP) chimera using baculovirus transgenes to MoDCs. **B)** The mean of fluorescent intensities ±standard deviation of LAMP1 were quantified in three different experiments (* *p*<0.001). **C)** The upper scattered plot shows the co-localization of red and green signals in sections of MoDCs infected with Pg381 and mutants strains. The lower plot shows the average of Pearson’s correlation measures of three randomly selected region of interest (ROI) within each field within 3 different experiments (* *p*<0.0001) (Table 4 and 5). All analysis of fluorescence intensity used One-way ANOVA analysis of different groups and Tukey’s test for multiple comparisons.

Due to the important role of lysosome fusion in autophagosome maturation [40], CFSE-labeled *P. gingivalis* and LAMP1+ lysosomes were tracked in MoDCs by epifluorescence microscopy and *P.gingivalis*-LAMP1 co-localization was quantified between 2 and 24 hours. The uptake of all three fimbriated *P. gingivalis* strains by MoDCs at 2, 12 and 24 hours relative to uninfected controls was confirmed. (Fig. 10A and 10B) (S8 Fig.). At 24 hours, Pg381 was mostly undetectable compared to Mfa1^+^Pg within MoDCs (S8 Fig.). Uptake of Pg381 was associated with increased LAMP1 at 2 hours, while minimal LAMP1 was detected in cells infected with Mfa1^+^Pg at all time points (S8 Fig.). The highest LAMP1 signal in Pg381 and FimA^+^Pg infected MoDCs was observed at 12 hours, yet there was no increase in LAMP1 signal within Mfa1^+^Pg infected MoDCs (Fig. 10A). Quantification of LAMP1 intensities within Pg381 and FimA^+^Pg -infected MoDCs was higher than Mfa1^+^Pg-infected and un-infected MoDCs (*p*<0.001) (Fig. 10B). In addition, co-localization was higher in Pg381 and FimA^+^Pg with LAMP1 at 12 hours (Fig. 10C) (Table 4 and 5).

**Table 4:** Co-localization of *P. gingivalis* with LAMP1 signals.

*P. gingivalis*		Mander’s overlap			Co-localization	
	Pearson’s correlation		coefficients		coefficients	
		Overlap	k1	k2	c1	c2
Pg381	0.7932	0.7579	0.3956	1.5187	0.9905	0.8350
Mfa1^+^Pg	-0.0159	0.3716	0.6256	0.2207	0.6090	0.9450
FimA^+^Pg	0.7719	0.8453	1.8998	0.2062	0.9680	0.9990

Quantifications of the fluorescent co-localizations within infected cells were done by NIS-Elements AR software. Three randomly selected regions of interest were selected for each field to quantify fluorescence dye intensities and co-localization.

**Table 5:** Pearson’s co-localization of LAMP1 and *P. gingivalis* signals.

Samples	Mean Diff.	95% CI of diff.	Adjusted P-Value
Pg381 vs. Mfa1^+^Pg	0.8117	0.6645 to 0.9588	*< 0.0001
Pg381 vs. FimA^+^Pg	0.1068	-0.0403 to 0.2540	0.1446
Mfa1^+^Pg vs. FimA^+^Pg	-0.7049	-0.852 to -0.5577	*< 0.0001

Pearson’s correlation of LAMP1 and *P. gingivalis* fluorescence (n = 3). Readings were analyzed by One-way ANOVA of different groups and Tukey’s test for multiple group comparisons (* statistical significance at the *p<0.01*)

## Discussion

Our results indicate that canonical autophagosomal and lysosomal clearance of *P. gingivalis* within DCs is dependent on initial routing to early endosomes, followed by autophagosomal and lysosomal routing. We show that *P. gingivalis* is able to survive within DCs by subversion of this canonical pathway via its Mfa-1 fimbriae. DCs are unlike macrophages and neutrophils in that the lysosomal machinery is specialized for efficient epitope preservation, rather total degradation [41]. Nevertheless, DCs must have mechanisms to control and inhibit the growth of intracellular pathogens; otherwise DCs could serve as a significant niche for pathogen dissemination to distant organs as recently suggested [42]. Autophagy has been widely recognized as an antibacterial lysosomal mechanism with an immune regulatory component [2,4,43][1]. Although the configuration of autophagy apparently serves DCs well in most situations, very little is known about its role in elimination of specific intracellular pathogens. More specifically, it is unclear how engagement of different PRRs on human myeloid DCs influences the induction of and maturation of antimicrobial autophagosomes.

The minor Mfa1 fimbriae are 67-kDa glycoproteins that target DC-SIGN for entry into MoDCs (and Raji cells) [14]. As this strain does not express FimA, a TLR2 agonist [28] expressed by all the other fimbriated strains, the present work represents a unique opportunity to study the role of PRRs on DCs in the context of antimicrobial autophagy. DC-SIGN is a type II transmembrane receptor that recognizes a wide range of pathogens through internal mannose branched structures and terminal di-mannose n-oligosaccharides [44]. Three internalization motifs (di-leucine motif, tri-acidic cluster, ITAM motif) on its cytoplasmic tail facilitate pathogen uptake [45]. Despite the diverse immune functions and regulations mediated by DC-SIGN [15,23,46,47], the immune escape mechanisms associated with DC-SIGN engagement by pathogens remain largely “unexplained” [17,18,20,21].

Our results are consistent with a role for DC-SIGN in routing *P. gingivalis* into distinct intracellular vesicles that escape early autophagosomal recognition and subsequent vesicle maturation and degradation. This DC-SIGN dependent re-routing seems to begin at the stage of early phagosomal formation, whereas DC-SIGN independent routing ‘traps’ *P. gingivalis* within Rab5 rich vesicles immediately after uptake. Rab5 has been suggested as an early stage initiator of autophagy and facilitator of subsequent lysosomal fusion [37][40]. In addition, Rab5 is reported to enhance autophagy by inhibition of mTORC1 (mTOR complex). The results of the current study suggest that when TLR signaling is weak (e.g. in the absence of FimA on the Mfa1^+^Pg strain) engagement of DC-SIGN dominates the response. This was clarified by adding exogenous TLR1/2 agonist, which inhibited Mfa1^+^Pg survival in parallel with higher LC3-II signals and stronger DC maturation. We surmise that lacking a strong signal for autophagy activation such as a TLR2 agonist, pathogens internalized by DC—SIGN are preferably routed to non-autophagosomal, non-lysosomal compartments where they survive in DCs.

One of the striking observations of the present work was the preferential routing of Mfa1^+^Pg to single-membrane intracellular vesicles, while the other strains were predominantly contained within characteristic double membrane phagophores. Classically, autophagy has been identified by the formation of double membrane vesicles that ultimately fuse with the lysosome for degradation of its intracellular components [48]. However, recently an alternative pathway known as non-canonical autophagy or LC3 associated phagocytosis (LAP) has been described [49], which involves the formation of single membrane vesicles. Here, DC-SIGN engagement appears to facilitate containment within single membrane vesicles; thus allowing *P. gingivalis* to evading lysosomal fusion. Some reports suggest that certain pathogens use (single membrane) autophagosomes for replication by blocking autophagosomal maturation [2,50,51]. Whether *P. gingivalis* utilizes DC-SIGN to enter a non-canonical pathway of autophagy for bacterial survival in DCs remains to be determined. One of the earliest studies describing *P. gingivalis* evasion of the conventional endocytic pathway and rerouting to (canonical) autophagosomes was in human aortic endothelial cells in [50]. Endothelial cells have been reported to express DC-SIGN [52] but this was not addressed in the previous study [50]. DCs, in contrast to endothelial cells, are highly migratory professional antigen presenting cells. Most reports of autophagy as an anti-microbial mechanism are from studies of immune cells [1,2,50,51,53]. In the present study of myeloid DCs, *P. gingivalis* appears to use an alternative tactic to evade autophagic capture through early engagement with DC-SIGN receptor and through non-canonical autophagy.

Analyses of intracellular survival of the different *P. gingivalis* strains suggest that DCs can use autophagy and lysosomal fusion to clear this intracellular pathogen. Pg381, which expresses both Mfa1 and FimA, engages both DC-SIGN and TLR2. It appears that DC-SIGN dependent routing is overruled by a strong TLR signal since Pg381 activates the autophagic process and is subsequently cleared, although this strain still survives longer inside MoDCs than outside in an aerobic atmosphere. Indeed, TLR signaling is a well-accepted inducer of autophagy as well as of phagosomal maturation [31–33]. However, the majority of autophagy studies that addressed TLRs were conducted in macrophages and neutrophils, both of which are naturally equipped with strong intracellular killing arsenal and higher expression of TLRs.

DC-SIGN expression is considered restricted to immature DCs with few exceptions in endothelium and specific macrophage subpopulations [54,55]. Our current data shows that DC-SIGN engagement may cause a positive feedback loop that increases the receptor expression resulting in subsequent bacterial uptake and survival. Increases in DC-SIGN+DCs in tissues of patients with periodontitis has previously been reported [56]. This could further enhance the pathogenicity of *P. gingivalis* and other DC-SIGN targeting pathogens. In the case of *P. gingivalis*, its ability to regulate fimbrial expression in different environmental cues such as pH, temperature and hemin content [26,27,34] may aid in its pathogenicity during chronic inflammation by regulation of DC-SIGN engagement (and expression). Current efforts in our laboratory are directed to identifying the levels of expression of Mfa1 and FimA in clinical samples of disease vs. healthy patients.

Phagosomes are known to have a high degree heterogeneity and individuality depending on host cell types, microbe captured and pattern recognition receptors engaged [11]. When *Staphylococcus aureus and Salmonella typhimurium* engage TLRs, for example, they are delivered to lysosomes at an inducible rate manifest by increased clearance and phagolysosomal fusion. However, slower phagolysosme maturation results from engagement with members of C-type lectin family and scavenger receptors [57]. DCs infected through DC-SIGN maintain an immature state and are more resistant to apoptosis [58]. Typically, immature DCs have a short life and active apoptosis is initiated shortly after maturation to avoid immune overstimulation [59,60]. Dominant DC-SIGN engagement alters such homeostatic balance and hinders the intracellular resistance to such infection. The DC-SIGN route may be a hallmark of chronic inflammation in response to low grade infection as it provides a protective niche for microbial persistence within the host. It is also influential in depolarizing the immune effector response [14]. Several lines of evidence have emerged linking autophagy and chronic inflammatory diseases [1,61,62]. In immature primary DCs, autophagy induction by NOD2 was essential for routing bacteria to lysosome and MHC presentation [61]. Early recognition of the microbe, here by DC-SIGN, could be crucial in driving ‘normal’ versus ‘up-normal’[1] autophagy and affect the fate of inflammatory process in chronic periodontitis.

The relevance of this work from a clinical standpoint comes from evidence that Mfa1+ *P. gingivalis* strains infect myeloid DCs in oral mucosal tissues and in blood of humans with periodontitis, wherein it is disseminated to distant sites of angiogenesis [42]. We conclude that this intracellular pathogen can survive within DCs by evasion of autophagy through coordinated regulation of Mfa1 and FimA expression. This may also facilitate its dissemination [42]. Potential therapeutic tactics for resolving chronic inflammatory diseases by forced autophagy to activate a strong immune response is also suggested by these studies.

## Materials and Methods

### Monocyte-derived DCs (MoDCs)

Human monocytes were isolated from mononuclear fractions of peripheral human blood by Human monocyte enrichment technique. After incubating the blood with the enrichment kit (RossetteSep, Cat. no. 15028) for 20 minutes, monocyte separation was carried out using medium density Ficoll (GE Healthcare, Cat. no. 17–1440–03). Cells were seeded in the presence of GM-CSF (1000 unit/ml, Gemini Bio-Product, Cat. no. 300–124P) and IL-4 (1000 unit/ml, Gemini Bio-Product, Cat. no. 300–154P) at a concentration (3–4 x 10^5^ cells/ml) for 5–6 days. Flow cytometry analyses were carried out to verify the immature DC phenotype (CD1a^+^, CD83^-^, CD14^-^, DC-SIGN^+^). Cell surface markers of DCs were evaluated by four-color immunofluorescence staining with the following antibodies: CD1a-PE (Miltenyi, Cat. no. 120–000–889), DC-SIGN-FITC (Miltenyi, Cat. no. 130–092–873), CD14-PerCP (Miltenyi, Cat. no. 130–094–969) and CD83-APC (Miltenyi, Cat. no. 130–094–186). After 30 min at 4°C and washing with staining buffer (PBS pH 7.2, 2 mM EDTA, and 2% FBS), cells were fixed in 1% paraformaldehyde. Positive marker expression was calculated as a percentage of total DCs by forward scatter and side scatter characteristics [14,23].

### Raji and Raji DC-SIGN^++^ (DCS) cells

To corroborate the immunoelectron microscopy staining of MoDCs for DC-SIGN, stably transfected DC-SIGN-positive (Raji-DCs) and negative Raji cells (Raji) were be obtained and the phenotype verified by flow cytometry[47]. The cells were cultured in 10% heat-inactivated FBS (Gemini, Cat. no. 100–500), RPMI 1640 with L-glutamine, and NaHCO3 (Cornning, Cat. no. 10041CM) in a 5% CO2 incubator at 37°C. Cells were centrifuged into a pellet and prepared for transmission electron microscopy sectioning.

### MoDCs treatment with HIV gp120 and Cytochalasin D

MoDCs were pre-incubated with HIV-1 gp120 Chiang Mai (CM) envelope protein (GP120) for 30 min at 37°C. GP120 protein was obtained through the National Institutes of Health AIDS Research and Reference Reagent Program, Division of AIDS, National Institute of Allergy and Infectious Diseases, National Institutes of Health (Cat. no. 2968) For actin polymerization inhibition, MoDCs were treated with cytochalasin D at 0.5 μM, the minimal concentration needed to arrest cytoskeletal rearrangements in Raji cells [14]. Cells were then washed 2 times with PBS and co-cultured with CFSE- stained *P. gingivalis* for 2, 12 and 24 hours at 37°C. Cells were fixed with 1% paraformaldehyde and prepared for immunofluorescence staining and epifluorescence microscopy.

### DC-SIGN Silencing in MoDCs

Cells were incubated with predesigned Siliencer Select siRNAs for DC-SIGN (Cat. No. 4392420 Ambion) for 24 hours at 10 nM concentration. 12 ul of lipofectamine 2000 Reagent (Cat. No. 11668–500 invitrogen) were used with Opti-MEM medium (Cat. No. 11058–021 LifeTechnologies) were used for siRNA delivery. Flow cytometry analysis was performed in control and infected MoDCs to confirm inhibition of DC-SIGN.

### Induction of autophagy

MoDCs were incubated with Rapamycin (Cat. no. Tlrl-rap, InvivoGen, San Diego, CA) at 200nM one hour after *P. gingivalis* infections. Induction of autophagy was confirmed by fluorescence staining of LC3-II within MoDCs after 2 and 6 hours.

### TLRs signaling

*E. coli* 026:B6 LPS (L2654, 2.5% protein, 1,500,000 EU/mg LPS, Sigma-Aldrich, St. Louis, MO). For stimulations, cells were treated with LPS at 1000 u/ml (200 ng/ml). For TLR1/2 stimulation Pam3CSK4 (Synthetic triacylated lipoprotein) (Cat. No. tlrl-pms, InvivoGen, San Diego, CA) were used at 1ug/ml.

### *Porphyromonas gingivalis* strains

Four *P. gingivalis* strains were used in this study; 1) Pg381, which expresses both minor (Mfa1) and major (FimA) fimbriae, 2) isogenic minor fimbria-deficient mutant (FimA^+^Pg), which expresses only the major fimbriae, 3) isogenic major fimbria-deficient mutant (Mfa1^+^Pg), which expresses only the minor fimbriae and 4) the double fimbriae mutant (MFB) (Table 1). *P. gingivalis* strains were maintained anaerobically in (10% H_2_, 10% CO_2_, and 80% N_2_) in a Forma Scientific anaerobic system glove box model 1025/1029 at 37°C in Difco anaerobe broth MIC [63]. Mutant strains were maintained using erythromycin (5 μg/ml) for mutant Mfa1^+^Pg, tetracycline (2 μg/ml) for mutant FimA^+^Pg and both erythromycin and tetracycline for double fimbriae mutant MFB.

### DC infection with *Porphyromonas gingivalis*

Bacteria suspensions were washed five times in PBS and re-suspended for spectrophotometer reading at OD 660 nm of 0.11, which previously determined to be equal to 5 x 10^7^ CFU [64]. For bacterial CFSE staining, the suspension were washed (3 times) and re-suspended in 5μM of CFSE in PBS. The bacteria were incubated for 30 min at 37°C in the dark [14,65]. MoDCs were pulsed with Pg381, Mfa1^+^Pg, FimA^+^Pg and MFB at 0.1, 1 and 10 MOI and incubated with the MoDCs for 2, 6, 12 and 24 hours and each experimental condition were performed in triplicate.

### Intracellular content of *P. gingivalis* strains within MoDCs

After 24 hours of MoDCs infection with *P. gingivalis* strains, cells were washed three times in PBS and re-suspended in sterile water on ice for 20 min to lyse the cells. Lysates were re-suspended in anaerobe broth for 3 days. After broth incubation, bacterial suspensions were washed three times in PBS and re-suspended for spectrophotometer reading at OD 660 in triplicate. Viable counts (CFU) were calculated based on a plate count serial dilution versus OD readings. For confirming the identity of the *P. gingivalis* (black pigmented Gram negative coccobacilli) suspensions were cultured on 5% blood agar plates in triplicate under anaerobic conditions (10% H2, 5% CO2 in nitrogen). Plates were incubated in anaerobic conditions at 35°C for 14 days until black colonies were detected and select colonies gram-stained.

### Survival assay of *P. gingivalis* in human MoDCs

After MoDCs were infected with the *P. gingivalis* strains for 2, 6, 12, 24 and 48 hours, cells were washed three times in PBS and re-suspended in sterile water on ice for 20 min to lyse the cells. Bacterial suspensions were washed three times in PBS and re-suspended for spectrophotometer reading at OD 660 in triplicate. Corresponding CFU counts were calculated based on a linear regression of plate count in serial dilution versus OD readings. Black colonies were confirmed in blood agar plate under anaerobic conditions (10% H2, 5% CO2 in nitrogen).

### Transmission electron microscopy (TEM)

After MoDCs fixation, the procedures were carried out at the Electron Microscopy and Histology Core, Department of Cellular Biology and Anatomy, Georgia Regents University. The cells were fixed in 2% glutaraldehyde in 0.1 M sodium cacodylate (NaCac) buffer, pH 7.4, postfixed in 2% osmium tetroxide in 0.1 M NaCac, stained en bloc with 2% uranyl acetate, dehydrated with a graded ethanol series and embedded in Epon-Araldite resin. Thin sections were cut with a diamond knife and stained with uranyl acetate and lead citrate. Cells were observed in transmission electron microscope (JEM 1230—JEOL USA Inc.) at 110 kV and imaged with a CCD camera and first light digital camera controller (Gatan Inc.).

### Immunogold transmission electron microscopy (IEM)

The procedures were carried out at the Electron Microscopy and Histology Core, Department of Cellular Biology and Anatomy, Georgia Regents University. Staining for DC-SIGN using anti-CD209 (mouse monoclonal, R&D Systems, # MAB161), was carried out to identify the *P. gingivalis*-containing vesicles. After DCs were pulsed with different *P. gingivalis* strains for the 2, 12 and 24 hours, cells were centrifuged into a pellet. Cells were be fixed in 4% formaldehyde 0.2% glutaraldehyde in 0.1 M sodium cacodylate (NaCac) buffer, pH 7.4, dehydrated with a graded ethanol series through 95% and embedded in LR White resin. Thin sections were cut with a diamond knife on a Leica EM UC6 ultramicrotome (Leica Microsystems Inc.) and collected on nickel grids. Sections were incubated in blocking buffer (5% BSA, 3% normal serum, 0.05% Tween-20 in Tris-buffered saline, pH 7.4) at room temperature in a humid chamber for 2 hours and with primary antibody diluted in blocking buffer overnight at 4°C. Grids were washed and incubated with gold-labeled secondary antibody for 2 hours at room temperature then washed and stained with 2% alcoholic uranyl acetate and 0.08% alkaline bismuth subnitrate. Cells were observed in a JEM 1230 transmission electron microscope (JEOL USA Inc.) at 110 kV and were imaged with an UltraScan 4000 CCD camera & First Light Digital Camera Controller (Gatan Inc.)

### Quantitative real time PCR

For RNA isolation, direct lysis of the cell suspensions were achieved by RNeasy kit (Cat. no. 74104, Qiagen) by adding 300 μl of Qiagen’s buffer RLT per sample. The lysates were collected and pipetted directly into the Qiashredder spin column. Ethanol (70%) was added and then samples were transferred to RNeasy spin columns. The samples were washed with buffer RW1, RBE, then the RNA samples were collected and stored at -80°C. RNA quantity and integrity were tested and only ratios of absorbance at 260 and 280 nm of 1.8–2.0, were included in the study. One-step qrt-PCR were performed using Express qPCR SuperMix (Cat. no. A10312, Invitrogen). Pre-formulated individual TaqMan gene expression primers (Applied Biosystems) were used for DC-SIGN mRNA detection (Hs.01588349_m1). For qrt-PCR reactions, 5μl of the RNA sample, 25μl PCR master mix (2x) and 2.5μl TaqMan gene expression assay were used per reaction. All PCRs were performed in triplicate and were carried out on a real-time PCR, StepOne (Applied Biosystems). For calculations and statistical analysis, fold changes were calculated using (2^-ΔΔCT^) method in the experimental samples [66]. Statistical analysis for gene expression was performed using the one sample t-test, which estimates the calculated difference (in fold-regulation) between experimental and control samples. A *p value* of <0.05 is the cut-off for significant differences.

### Flow cytometry of pattern recognition receptors (PRRs)

Cells were fixed with 4% paraformaldehyde, blocked and counterstained with fluorescent-labeled antibodies against Anti-Human CD206 (MMR) (Cat. No. 53–2069–41), Anti-Human DCIR (Clec4A) (Cat. No. 12–9875–41), Anti-Human CD209 (DC-SIGN) (Cat. No. 45–2099–41), Anti-Human Dectin-1 (Cat. No. 46–9856–41), Anti-Human CD284 (TLR4) (Cat. No. 12–9917–41), Anti-Human CD184 (CXCR4) (Cat. No. 15–9999–41), Anti-Human CD282 (TLR2) (Cat. No. 17–9922–41), Anti-Human CD286 (TLR6) (Cat. No. 13–9069–80) (all ebioscience, USA). All markers were measured against isotype controls. The markers were measured as MFI using the Accuri C6 Flow Cytometry system.

### LC3-II immuno-fluorescence and epifluorescence microscopy

MoDCs were infected with *P. gingivalis* prelabeled with carboxyfluorescein succinimidyl ester (CSFE) fluorescence. Cells were fixed with 1% paraformaldehyde, washed with PBS twice and permeabalized with 0.5% saponin. MoDCs were incubated with LC3-II antibody (ab51520) for 2 hours and then washed with PBS. Pellets were res-suspended in cytospin fluid (Cat. no. 6768315, Shandon) centrifuged at 400 rpm for 4 minutes. Slides were mounted with anti-fade reagent (Invitrogen, P36931) and dried for microscopic analysis. Microscopic images were obtained with epifluorescence microscope (Nikon E600) then analyzed by image enhanced fluorescence microscopy aided by deconvolution analysis. Quantifications of the fluorescent intensities and co-localization within infected cells were done by NIS-Elements BR and AR software. Three randomly selected regions of interest were selected for each field to quantify fluorescence dye intensities.

### Western blot analysis

Cells were centrifuged and washed twice with PBS. After washing, cells were lysed by addition of cell lysis buffer (Cell signaling Cat. no. 9803S) and incubated for 20 minutes on ice. Samples were centrifuged and the supernatant was collected and stored at -80°C. Proteins were denatured at 70°C for 10 minutes immediately prior to loading. For immunoblotting, 50 μg of total cellular protein per lane were separated by blot 4% to 12% Bis Tris Plus gradient gel and transferred to PVDF (polyvinylidine difluoride) membranes using iBlotting dry transfer system (Lifetechnologies, Cat. no. IB1001). The membranes were incubated with primary antibody LC3B (Abcam, Cat. No. ab48394) or GAPDH (Meridian life science, Cat. No. H86504M) and secondary antibody peroxidase-conjugated goat anti-rabbit or goat anti-mouse IgGs in iBind solution for 2.5 hours (iBind western system, life technologies). The specific protein signals were visualized using chemiluminescent peroxidase substrate and exposing the membranes to the high performance chemiluminescene film for detection. Protein loading was verified by detection of GAPDH using mouse anti-GAPDH monoclonal antibody.

### Detection of LAMP1, Rab5 and 7 via transduction of fluorescent protein chimera by baculovirus transgenes

Monocytes were transduced with CellLight BacMam 2.0 (lifetechnologies) to visualize lysosomal marker LAMP1 (C10504), early endosome Rab5 (C10587) and late endosome Rab7 (C10589). Transduction was performed simultaneously with differentiation of MoDCs at the 5th day. Cells were transfected with 30 PPC (particle per cell) for 24 hours. At the 6th day MoDCs were harvested and immature phenotype (CD1c^+^DC-SIGN^+^CD83^-^CD14^-^) by flow cytometry. Quantifications of the fluorescent intensities and co-localization within infected cells were done by NIS-Elements BR and AR software.

### Ethical aspects

These studies were determined by the Human Assurance Committee at Georgia Regents University to be human subject exempt, due to the use of anonymized peripheral blood samples for monocytes.

## Supporting Information

S1 Fig High intracellular content Mfa1^+^Pg within MoDCs after 24 hours of infection. Transmission electron microscopy (TEM) of MoDCs infected with *P. gingivalis* for 24 hours. The sections show the intra-and extra-cellular content of Pg381 (upper) and Mfa1^+^Pg (lower) at different magnifications.
(TIF)

S2 Fig The expression of DC-SIGN after *P. gingivalis* infection of human MoDCs. The figure shows DC-SIGN mRNA expression at 2, 6 and 24 hours of MoDCs infected at 0.1, 1 and 10 MOIs. The target gene (DC-SIGN) was normalized using the endogenous control GAPDH (ΔCt) and fold regulations were calculated using 2^-(ΔΔCt)^ method. The statistical analysis was performed using the t-test, which accounts for the clustering of infected and un-infected controls within 3 different experiments.
(TIF)

S3 Fig Immuno-electron Transmission Microscopy of Raji cells and MoDCs. **A)** Sections showed gold labeling of DC-SIGN (blue arrows) at 1/10, 1/20 and 1/50 dilutions of antibodies. Left panels showed the staining of Raji DC-SIGN—ve cells and Raji DC-SIGN+ve cells are on the right panels. **B)** Immuno-electron microscopy of un-infected MoDCs (Cont.) and cells infected with Pg381, Mfa1^+^Pg and FimA^+^Pg. Gold particles (black with red arrows) for positive DC-SIGN were detected in the cell membrane and cytoplasm of cells infected with Mfa1^+^Pg strains.
(TIF)

S4 Fig Flow cytometry analysis of different pattern recognition receptors (PPRs) on infected MoDCs. Cells infected with Pg38, Mfa1^+^Pg and FimA^+^Pg showed; decreased TLR6 and increased CXCR4 and TLR2 (*p<0.01)*. However, no distinction was detected between the different *P. gingivalis* strains (Pg381 and Mfa1^+^Pg) in the expression of TLRs. For the C-type lectin receptors, *P. gingivalis* infection increased the expression of DCIR and mannose receptor (MMR) *(p<0.01)*. However, the increase of DC-SIGN and Dectin were only detected in cells infected with Mfa1^+^Pg (*p<0.001)*. The statistical analyses were performed by One-way ANOVA of different groups and Tukey’s test for multiple group comparisons within 3 different experiments.
(TIF)

S5 Fig Uptake of *P. gingivalis* by MoDCs and expression of early and late endosomes. **A)** Epifluorescence microscopy images of MoDCs infected with pre-labeled bacteria (green-CFSE) for 2, 12 and 24 hours. Rab5 detected by transduction of red fluorescent protein (RFP) chimera using baculovirus transgenes to MoDCs. **B)** Fluorescent intensities ±standard deviation of Rab5 were quantified in three different experiments 2 to 24 hours. **C)** Epifluorescence microscopy images of Rab7 within MoDCs after 2, 12, 24 hours of infections with Pg381 and mutant strains. Rab7 was detected by transduction of red fluorescent protein (RFP) chimera using baculovirus transgenes and bacteria with green-CFSE. **D)** Fluorescent intensities ±standard deviation of Rab7 were quantified in three different experiments 2 to 24 hours. All analysis used Kruskal-Wallis test of different groups and Dunn’s test for multiple comparisons (*p<0.001).
(TIF)

S6 Fig LC3-II expression in MoDCs infected with *P. gingivalis* 2–24 hours. **A)** Epifluorescence microscopy images of LC3-II within MoDCs infected with Pg381 and Mfa1^+^Pg for 2, 6, 12 and 24 hours. **B)** Fluorescent intensities ±standard deviation of LC3-II were quantified in three different experiments (2 to 24 hours). All analysis used One-way ANOVA analysis of different groups and Tukey’s test for multiple comparisons (*p<0.001).
(TIF)

S7 Fig Bafilomycin dose response in human MoDCs A) The dose response of MoDCs to different concentration of Bafilomycin after 4 hours of treatment. **B)** LC3-II intensity in MoDCs treated with different concentrations of Bafilomycin. Accordingly (3nM) was used for treating MoDCs for autophagy flux test. **Inhibition of endocytosis blocks the uptake of Pg381 and Mfa1** ^**+**^ **Pg and restores basal LC3-II level. C)** Immuno-fluorescence images of LC3-II and *P. gingivlis* in MoDCs treated with Cytochalasin D (CytD). **D)** Graph shows the fluorescent intensity quantification of LC3-II (grey bars) and CFSE (black bars) in MoDCs. All analysis of fluorescence intensity used One-way ANOVA analysis of different groups and Tukey’s test for multiple comparisons (# *p*<0.001).
(TIF)

S8 Fig Low level of lysosomal marker (LAMP-1) in MoDCs after uptake of *P. gingivalis* by DC-SIGN receptor. **A) B)** Epifluorescence microscopy images of MoDCs infected with pre-labeled bacteria (green-CFSE). LAMP1 detected by transduction of red fluorescent protein (RFP) chimera using baculovirus transgenes to MoDCs. The sections shows the cells infected with Pg381 and Mfa1^+^Pg strains for 2 and 24 hours. C) Graph shows the fluorescent intensity quantification of LAMP within MoDCs 2 to 24 hours after infections. All analysis of fluorescence intensity used One-way ANOVA analysis of different groups and Tukey’s test for multiple comparisons (# *p*<0.001)
(TIF)
