# Supplementary figures and images for: Porphyromonas gingivalis Evasion of Autophagy and Intracellular Killing by Human Myeloid Dendritic Cells Involves DC-SIGN-TLR2 Crosstalk
Source: PLoS Pathog. 2015 Feb 13;11(2):e1004647. doi: 10.1371/journal.ppat.1004647 (PMC4352937; doi:10.1371/journal.ppat.1004647)

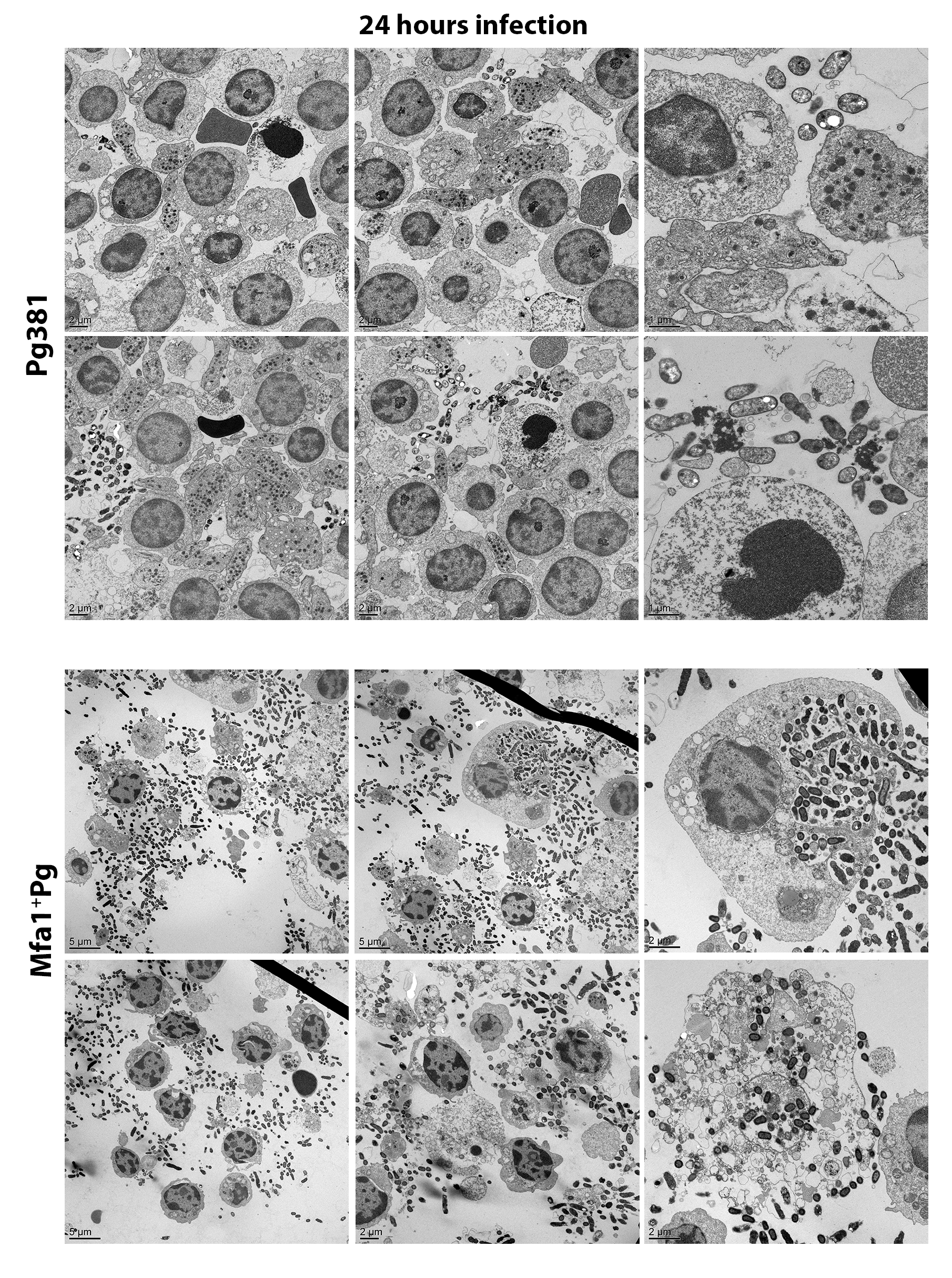

Supplement: S1 Fig — Transmission electron microscopy (TEM) of MoDCs infected with P. gingivalis for 24 hours. The sections show the intra-and extra-cellular content of Pg381 (upper) and Mfa1+Pg (lower) at different magnifications. (TIF) [file ppat.1004647.s001.tif]

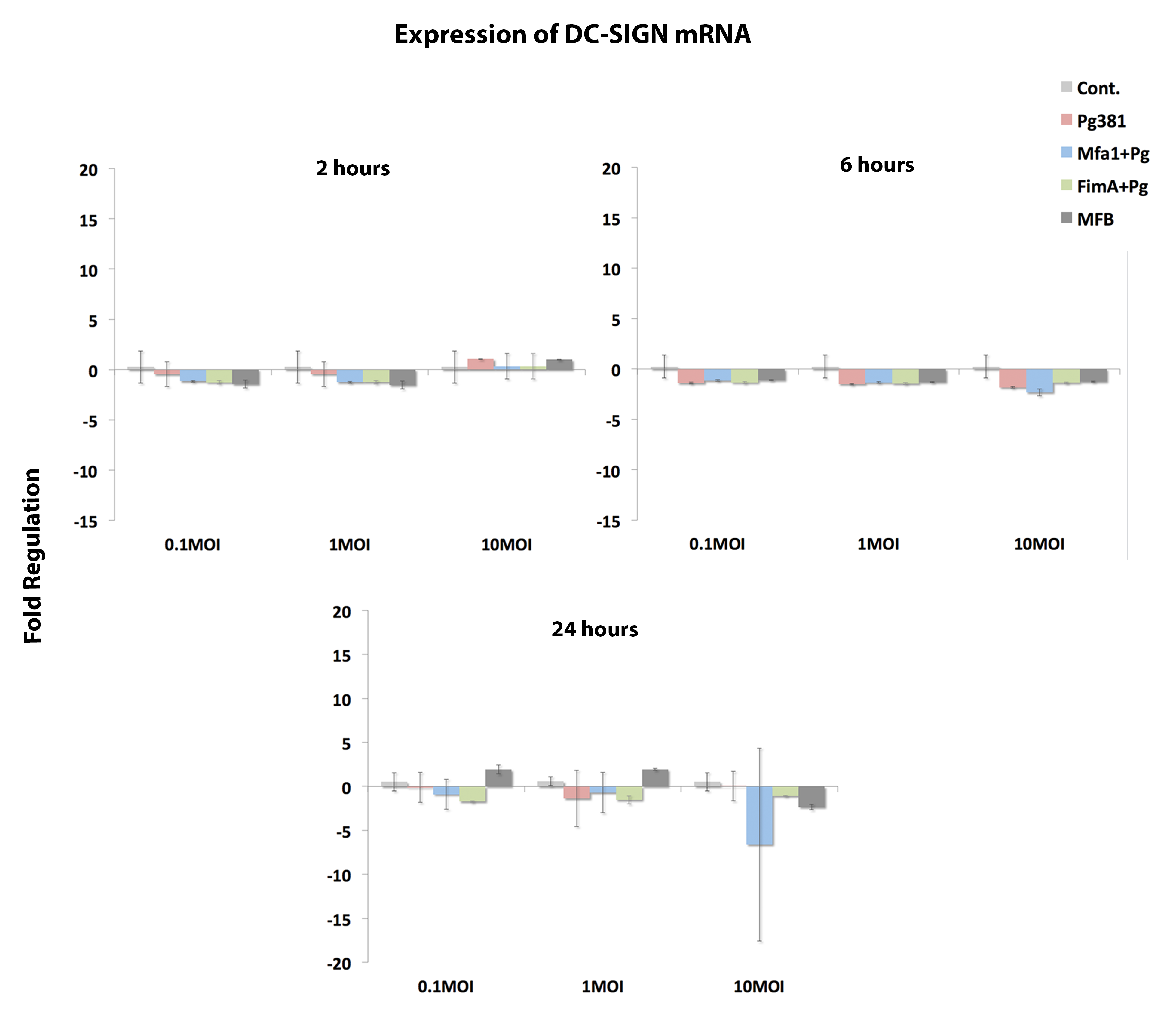

Supplement: S2 Fig — The figure shows DC-SIGN mRNA expression at 2, 6 and 24 hours of MoDCs infected at 0.1, 1 and 10 MOIs. The target gene (DC-SIGN) was normalized using the endogenous control GAPDH (ΔCt) and fold regulations were calculated using 2-(ΔΔCt) method. The statistical analysis was performed using the t-test, which accounts for the clustering of infected and un-infected controls within 3 different experiments. (TIF) [file ppat.1004647.s002.tif]

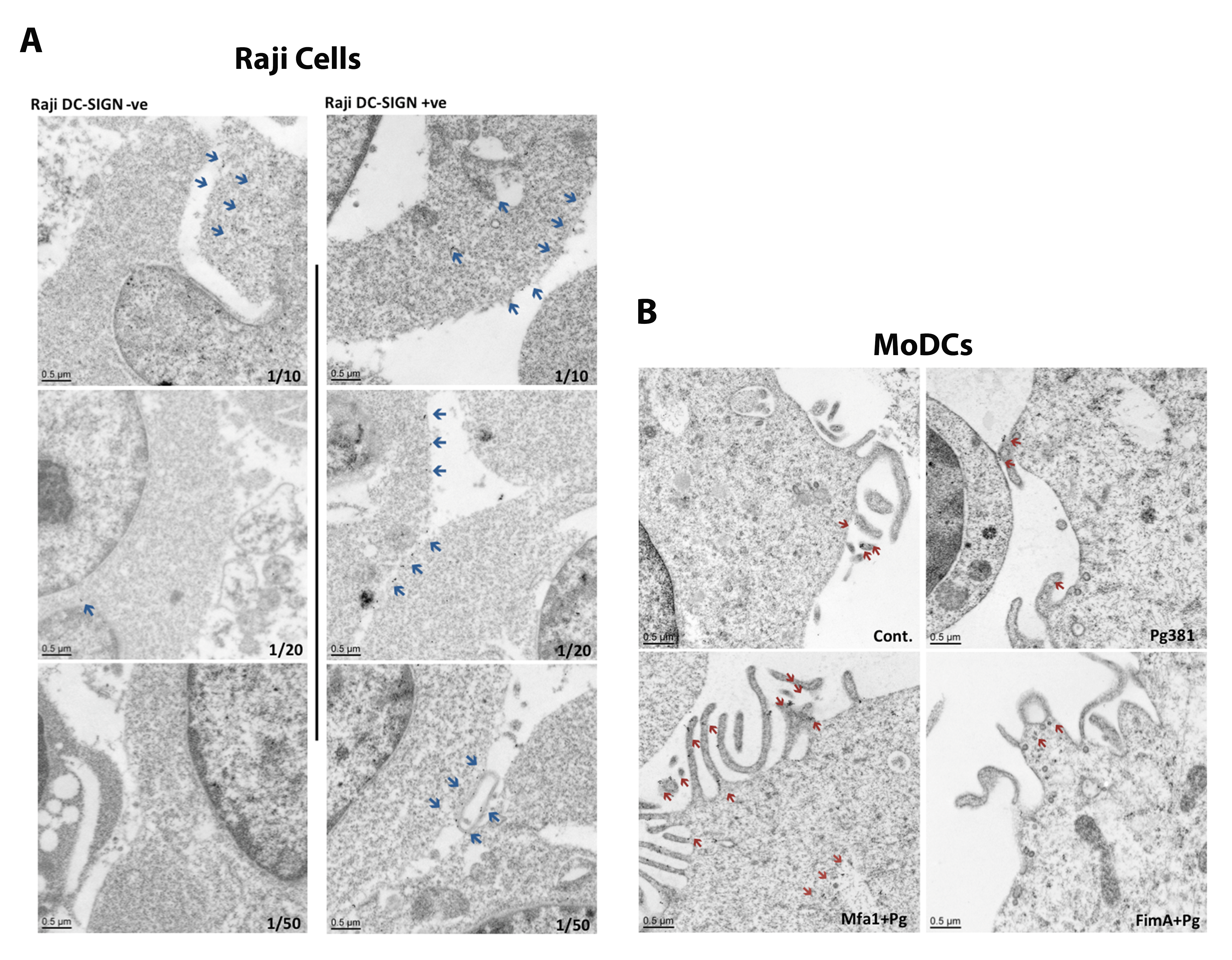

Supplement: S3 Fig — A) Sections showed gold labeling of DC-SIGN (blue arrows) at 1/10, 1/20 and 1/50 dilutions of antibodies. Left panels showed the staining of Raji DC-SIGN—ve cells and Raji DC-SIGN+ve cells are on the right panels. B) Immuno-electron microscopy of un-infected MoDCs (Cont.) and cells infected with Pg381, Mfa1+Pg and FimA+Pg. Gold particles (black with red arrows) for positive DC-SIGN were detected in the cell membrane and cytoplasm of cells infected with Mfa1+Pg strains. (TIF) [file ppat.1004647.s003.tif]

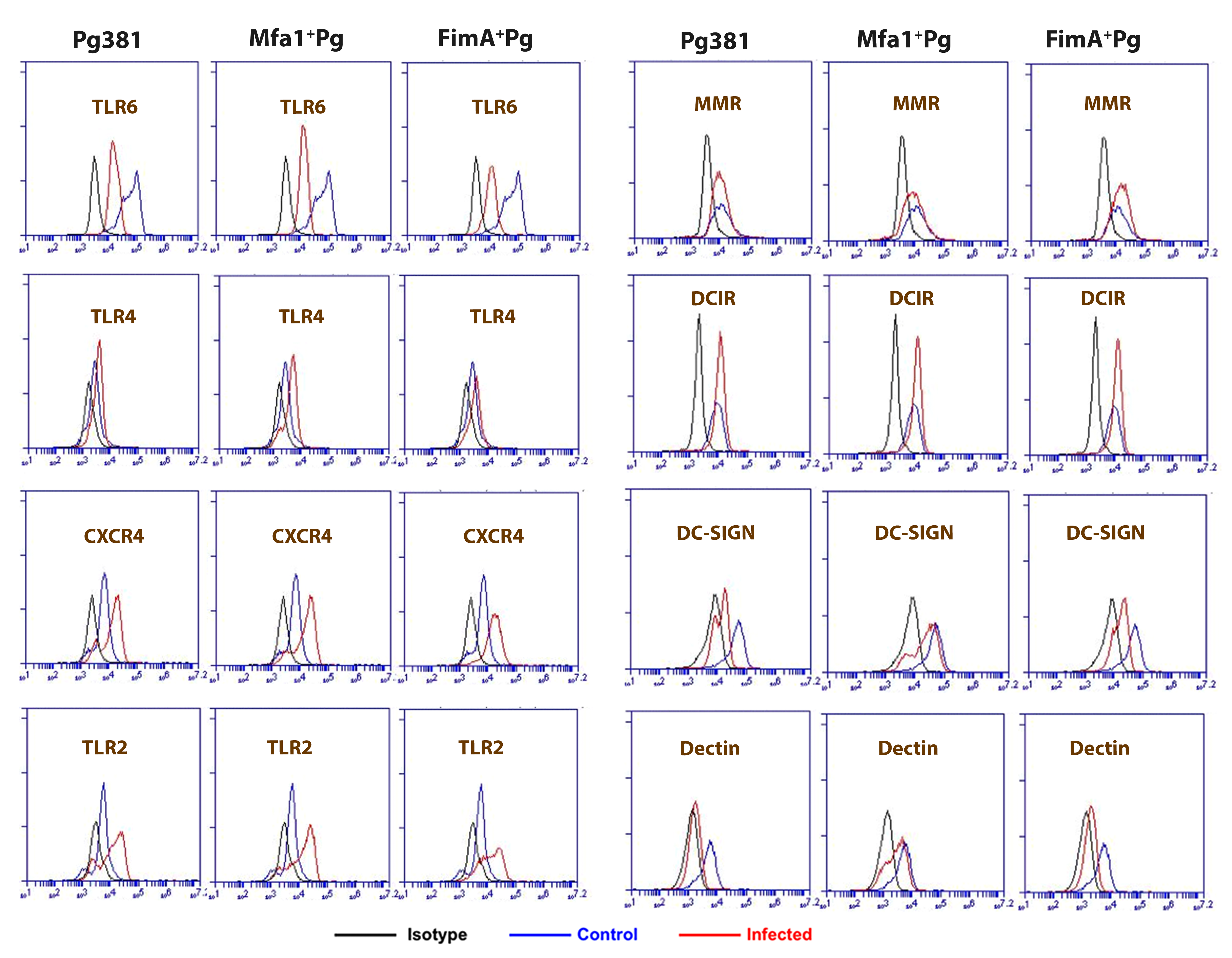

Supplement: S4 Fig — Cells infected with Pg38, Mfa1+Pg and FimA+Pg showed; decreased TLR6 and increased CXCR4 and TLR2 (p<0.01). However, no distinction was detected between the different P. gingivalis strains (Pg381 and Mfa1+Pg) in the expression of TLRs. For the C-type lectin receptors, P. gingivalis infection increased the expression of DCIR and mannose receptor (MMR) (p<0.01). However, the increase of DC-SIGN and Dectin were only detected in cells infected with Mfa1+Pg (p<0.001). The statistical analyses were performed by One-way ANOVA of different groups and Tukey’s test for multiple group comparisons within 3 different experiments. (TIF) [file ppat.1004647.s004.tif]

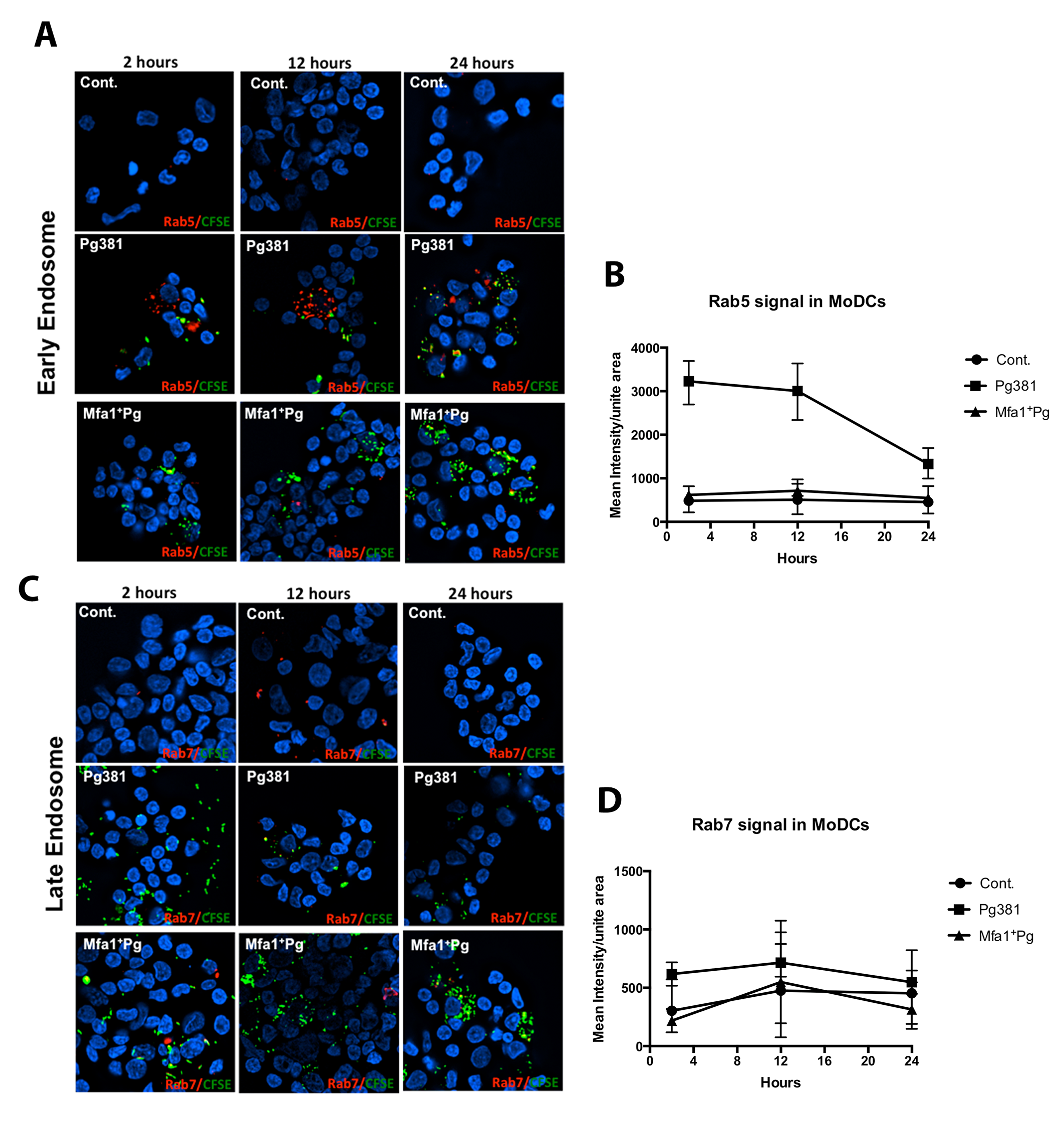

Supplement: S5 Fig — A) Epifluorescence microscopy images of MoDCs infected with pre-labeled bacteria (green-CFSE) for 2, 12 and 24 hours. Rab5 detected by transduction of red fluorescent protein (RFP) chimera using baculovirus transgenes to MoDCs. B) Fluorescent intensities ±standard deviation of Rab5 were quantified in three different experiments 2 to 24 hours. C) Epifluorescence microscopy images of Rab7 within MoDCs after 2, 12, 24 hours of infections with Pg381 and mutant strains. Rab7 was detected by transduction of red fluorescent protein (RFP) chimera using baculovirus transgenes and bacteria with green-CFSE. D) Fluorescent intensities ±standard deviation of Rab7 were quantified in three different experiments 2 to 24 hours. All analysis used Kruskal-Wallis test of different groups and Dunn’s test for multiple comparisons (*p<0.001). (TIF) [file ppat.1004647.s005.tif]

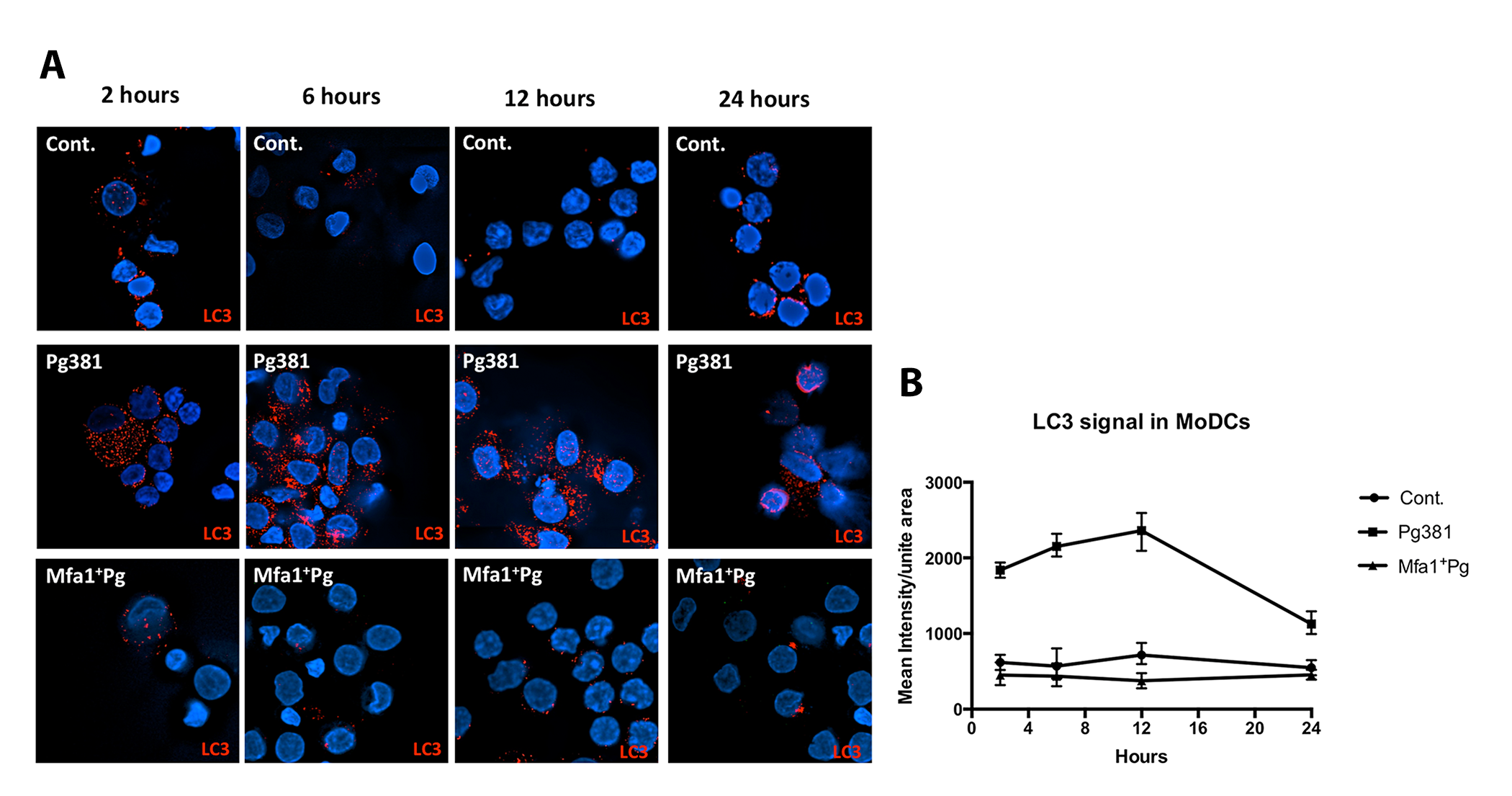

Supplement: S6 Fig — A) Epifluorescence microscopy images of LC3-II within MoDCs infected with Pg381 and Mfa1+Pg for 2, 6, 12 and 24 hours. B) Fluorescent intensities ±standard deviation of LC3-II were quantified in three different experiments (2 to 24 hours). All analysis used One-way ANOVA analysis of different groups and Tukey’s test for multiple comparisons (*p<0.001). (TIF) [file ppat.1004647.s006.tif]

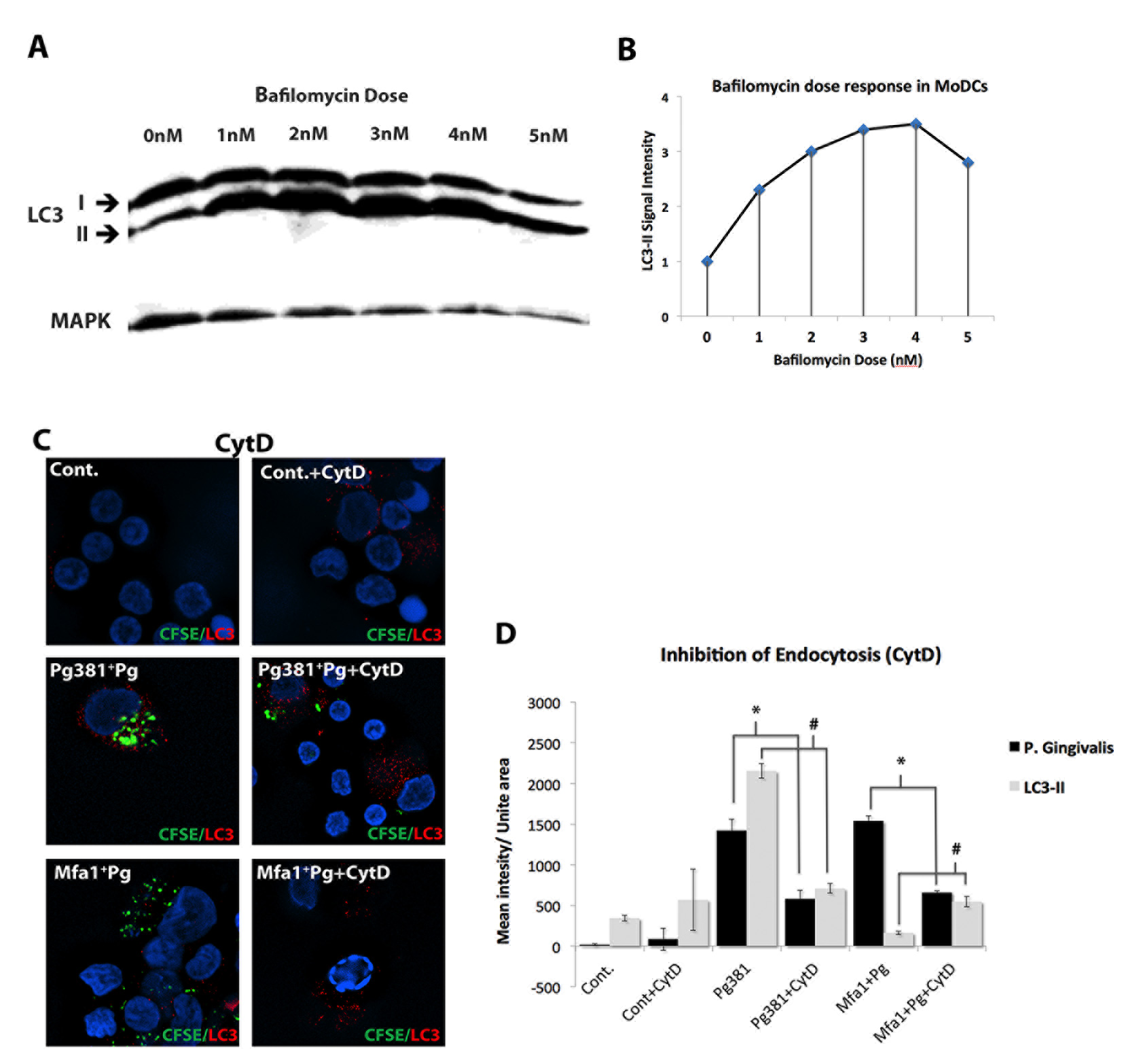

Supplement: S7 Fig — B) LC3-II intensity in MoDCs treated with different concentrations of Bafilomycin. Accordingly (3nM) was used for treating MoDCs for autophagy flux test. Inhibition of endocytosis blocks the uptake of Pg381 and Mfa1 + Pg and restores basal LC3-II level. C) Immuno-fluorescence images of LC3-II and P. gingivlis in MoDCs treated with Cytochalasin D (CytD). D) Graph shows the fluorescent intensity quantification of LC3-II (grey bars) and CFSE (black bars) in MoDCs. All analysis of fluorescence intensity used One-way ANOVA analysis of different groups and Tukey’s test for multiple comparisons (# p<0.001). (TIF) [file ppat.1004647.s007.tif]

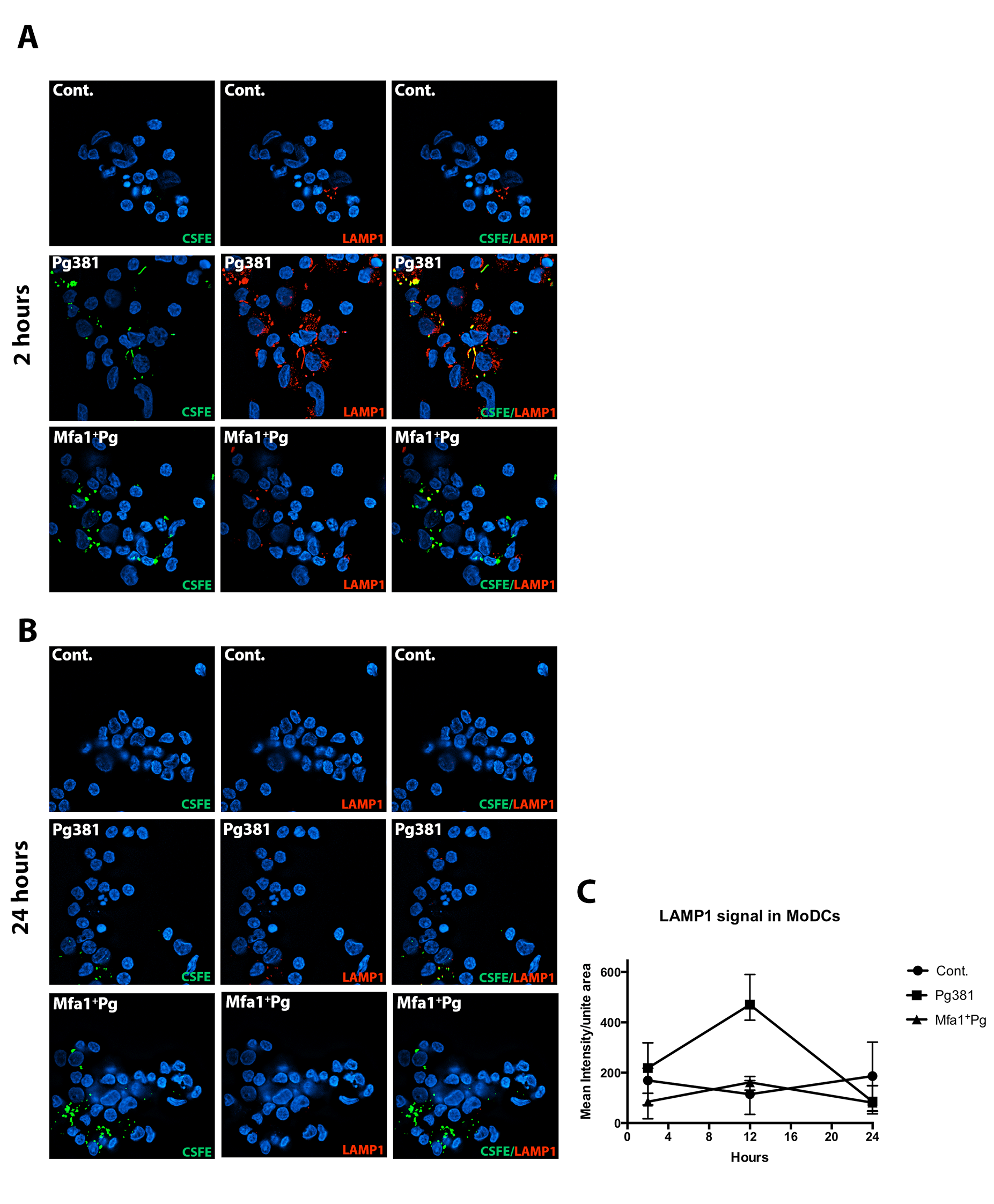

Supplement: S8 Fig — A) B) Epifluorescence microscopy images of MoDCs infected with pre-labeled bacteria (green-CFSE). LAMP1 detected by transduction of red fluorescent protein (RFP) chimera using baculovirus transgenes to MoDCs. The sections shows the cells infected with Pg381 and Mfa1+Pg strains for 2 and 24 hours. C) Graph shows the fluorescent intensity quantification of LAMP within MoDCs 2 to 24 hours after infections. All analysis of fluorescence intensity used One-way ANOVA analysis of different groups and Tukey’s test for multiple comparisons (# p<0.001) (TIF) [file ppat.1004647.s008.tif]
